# Effect of dynamic exclusion and the use of FAIMS, DIA and MALDI-mass spectrometry imaging with ion mobility on amyloid protein identification

**DOI:** 10.1186/s12014-024-09500-w

**Published:** 2024-07-03

**Authors:** Jennifer T. Aguilan, Jihyeon Lim, Sabrina Racine-Brzostek, Joshua Fischer, Cristina Silvescu, Shannon Cornett, Edward Nieves, Damodara Rao Mendu, Carlos-Madrid Aliste, Stacia Semple, Ruth Angeletti, Louis M. Weiss, Adam Cole, Michael Prystowsky, James Pullman, Simone Sidoli

**Affiliations:** 1 Laboratory for Macromolecular Analysis and Proteomics Facility, Albert Einstein College of Medicine, New York, 10461 USA; 2 Department of Pathology, Albert Einstein College of Medicine, New York, 10461 USA; 3 Montefiore Medical Center, Moses and Weiler Campus, New York, 10461 USA; 4 Department of Biochemistry, Albert Einstein College of Medicine, Bronx, NY 10461 USA; 5 Department of Systems and Computational Biology, Albert Einstein College of Medicine, New York, 10461 USA; 6 Janssen Research and Development, Malvern, PA USA; 7 Department of Pathology, Weill Cornell Medical Center, New York, USA; 8 Clinical Chemistry Laboratory, Mount Sinai School of Medicine, New York, USA; 9 Northwell Health Hospital, New York, USA; 10 Bruker Daltonics Corporation, New York, USA

**Keywords:** Amyloidosis, MALDI-MSI, Laser capture microdissection, Transthyretin amyloidosis, Light chain amyloidosis, DIA, DDA, FAIMS

## Abstract

Amyloidosis is a disease characterized by local and systemic extracellular deposition of amyloid protein fibrils where its excessive accumulation in tissues and resistance to degradation can lead to organ failure. Diagnosis is challenging because of approximately 36 different amyloid protein subtypes. Imaging methods like immunohistochemistry and the use of Congo red staining of amyloid proteins for laser capture microdissection combined with liquid chromatography tandem mass spectrometry (LMD/LC–MS/MS) are two diagnostic methods currently used depending on the expertise of the pathology laboratory. Here, we demonstrate a streamlined in situ amyloid peptide spatial mapping by Matrix Assisted Laser Desorption Ionization–Mass Spectrometry Imaging (MALDI-MSI) combined with Trapped Ion Mobility Spectrometry for potential transthyretin (ATTR) amyloidosis subtyping. While we utilized the standard LMD/LC–MS/MS workflow for amyloid subtyping of 31 specimens from different organs, we also evaluated the potential introduction in the MS workflow variations in data acquisition parameters like dynamic exclusion, or testing Data Dependent Acquisition combined with High-Field Asymmetric Waveform Ion Mobility Spectrometry (DDA FAIMS) versus Data Independent Acquisition (DIA) for enhanced amyloid protein identification at shorter acquisition times. We also demonstrate the use of Mascot’s Error Tolerant Search and PEAKS de novo sequencing for the sequence variant analysis of amyloidosis specimens.

**Supplementary Information:**

The online version contains supplementary material available at 10.1186/s12014-024-09500-w.

## Introduction

Amyloidosis is a rare disease caused by the anomalous accumulation of undegraded misfolded proteins that form fibrils in tissues from different organs. This leads to organ failure if left undiagnosed and untreated. Amyloidosis disease can be subtyped based on the amyloid protein that accumulates in different organs of the body. The International Society of Amyloidosis Nomenclature Committee has named and classified about 36 different amyloid protein subtypes that can be further characterized as systemic or local and wild type or familial type [1]. The nomenclature system for the disease uses a key comprised of the symbol “A” for Amyloidosis followed by the protein name. The diagnosis of this disease can be difficult due to the 36 different subtypes. Kappa or lambda light chain amyloidosis (ALκ or ALλ), transthyretin (ATTR) and Serum amyloid A-1 (AA) amyloidosis are the four most commonly known amyloidosis subtypes [2]. Age-associated senile wild type amyloidosis are diagnosed in patients who are ≥ 50 years old and although it is currently considered as a rare disease, the number of cases continue to increase over time [3–6]. On the other hand, hereditary forms of amyloidosis due to the amyloid protein sequence variants can be diagnosed early in the younger age groups [4, 7–9]. Diagnosis of hereditary amyloidosis becomes more challenging due to various mutations per amyloid protein subtype: transthyretin (TTR; > 100 variants), apolipoprotein A1 (APOA1; > 20 variants), apolipoprotein A2 (APOA2; 4 variants), fibrinogen alpha (FGA; > 9 variants), lysozyme (LYZ; 10 variants) and gelsolin (GSN; 4 variants) [10–16]. An estimate of the frequency of occurrence of amyloidosis was provided by a proteomics based study of a large cohort of 16,175 amyloidosis specimens (including hereditary amyloidosis) analysed from 2008–2018 showed AL amyloidosis with the highest frequency at 59.0%, followed by ATTR (28.4%), ALECT2 (3.2%), AA (2.9%), AH (2.3%) and the rest of the other types of amyloidosis were < 1.0% [17].

Due to the complexity of the treatment for amyloidosis [18–22], clinicians follow a rigorous diagnostic pipeline to identify the proper subtype. The diagnosis could first be attempted by using less or non-invasive approaches (e.g. echocardiography, magnetic resonance imaging, imaging of reactive tags injected into circulation, PET/CT or serum and urine immunoelectrophoresis) before resorting to more invasive tissue biopsy [21, 23–29]. Biopsy specimens are collected either in the form of fat pad aspirate or as formalin fixed paraffin embedded (FFPE) tissues [30]. The presence of amyloid proteins that form well-ordered fibril structures can be detected by positive staining with Congo red dye [31, 32]. Presence of Serum amyloid P component (SAP), Apolipoprotein E (APOE), Apolipoprotein A-IV (APOAIV) and glycosaminoglycans have been detected and described as markers associated with the amyloid proteins that form the fibril structures within the tissue [33, 34]. Congo red stained amyloid proteins produce a salmon pink color when viewed under bright field, apple-green birefringence under polarized light and fluorescent red under Texas red filter. Although Congo red staining detects the location of amyloid proteins on the tissue, it is non-specific to amyloid fibrils and is not capable of identifying the amyloid protein [32]. Subsequent steps after Congo red staining need to be done to diagnose the amyloidosis subtype by identifying the amyloid protein. Immunohistochemistry for the detection of amyloidosis has become the gold standard since this method is readily available in many pathology laboratories [35]. However, misdiagnosis of the amyloid protein subtype has been reported due to drawbacks that preclude correct amyloid protein subtyping due to non-specificity issues, missing epitopes due to truncated proteins, unavailability of antibodies and requirement for multiple serial sections to screen for amyloid protein subtype [36–39].

Mass spectrometry-based proteomics has become the alternative to immunohistochemistry and the other abovementioned methods for amyloid protein subtyping [21, 23, 28, 40, 41]. Mayo Clinic pioneered the amyloid protein identification by the use of combined laser capture microdissection and shotgun proteomics by liquid chromatography tandem mass spectrometry (LMD/LC–MS/MS) [30, 40, 42, 43]. Briefly, Congo red positive areas on the FFPE tissue section are sectioned and collected by laser capture microdissection. Proteins are then extracted, digested with trypsin and peptides are analyzed by LC–MS/MS. This technique eliminates the use of antibodies and offers multiple protein detection on a single tissue section. This method employs a data dependent type of acquisition (DDA) where the most intense precursor peptide ions are selected in the MS1 stage and then each one is fragmented one at a time to generate its own MS/MS fingerprint spectrum at MS2 stage where peptide sequence information is derived through automated protein database search. In contrast to DDA, data independent acquisition (DIA) fragments every precursor ions within a selected mass range resulting in a complex MS/MS spectra from co-fragmenting precursor ions which are deconvoluted by specific software that utilize mass spectral library match or library free approach [44–47]. Although DIA is at first more computationally challenging due to the mixed MS/MS spectra, there is growing interests in the development of DIA method for clinical assays due to the enhanced confidence in extracting peptide quantification [48–50]. The reason is that DIA-based runs can be analyzed in a similar manner to targeted acquisition data such as selected reaction monitoring (SRM) or by multiple reaction monitoring (MRM) [51–53]. Finally, matrix assisted laser desorption ionization-mass spectrometry imaging (MALDI-MSI) has also been used to do spatial mapping of amyloid proteins/peptides on either a flash frozen or FFPE tissue section [54–56].

**Fig. 1:**
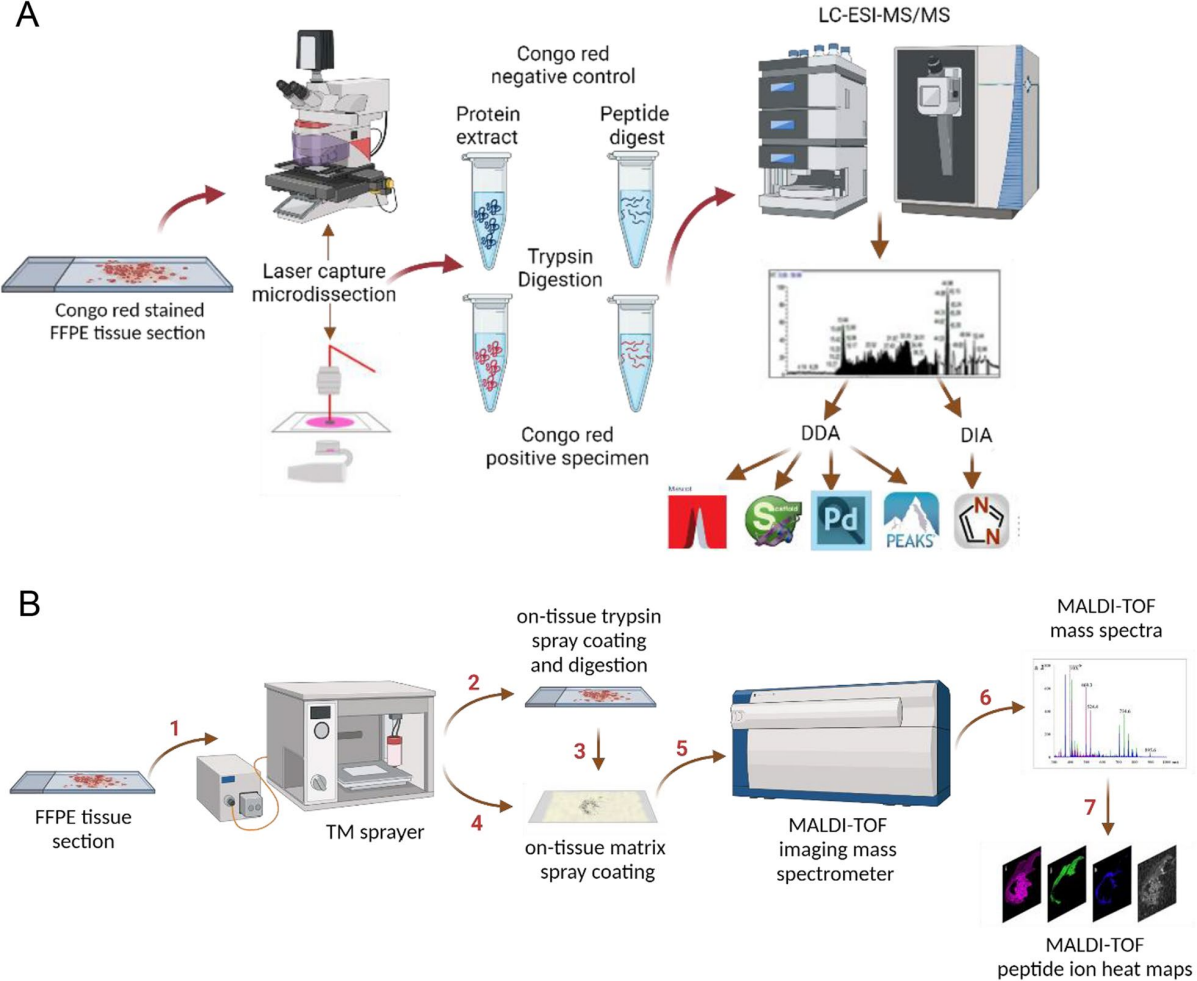
Schematic diagram of the workflow for amyloidosis analysis using **a** LMD/LC–MS/MS **b** MALDI-MSI

In this manuscript, we compared emerging techniques in proteomics as potential alternative or complementary tools for the analysis of amyloidosis specimens (Fig. 1). Specifically, we utilized LMD/LC–MS/MS in combination with FAIMS, DIA and Error Tolerant spectra identification and de novo sequencing for sequence variant identification. We also combined MALDI-MSI with Trapped Ion Mobility Spectrometry for amyloid tissue imaging.

## Methods

### FFPE human tissue samples

All FFPE human tissue samples were de-identified before conducting any analysis and this study was approved by the Institutional Review Board (IRB) of Albert Einstein College of Medicine.

### Amyloid protein subtyping by LMD/LC–MS/MS

FFPE tissue section (10 µm) mounted on polyethylene naphthalate (PEN) membrane slides were deparaffinized at 60 °C for 1.5 h, then washed through a series of xylene, 100% ethanol, 95% ethanol, 70% ethanol, water, and then stained with Congo red and counter-stained with hematoxylin. The Congo red positive areas on the tissue section were cut by a Leica ASLMD laser capture microscope and collected into the cap filled with extraction buffer (10 mM Tris, 2 mM EDTA, 0.002% Zwittergent 3–16) of a small PCR tube [43]. The tubes were centrifuged and heated to 95–98 °C for 90 min and sonicated for 1 h at 80 °C. The extracted proteins were digested with 500 ng of trypsin (Sigma Cat. No. T6567-5X20UG) for 16–18 h. The peptide digests were desalted using C-18 ziptip or Oasis HLB resin by vacuum filtration and eluted with 60% acetonitrile/0.1% TFA, concentrated in a speed vacuum and resuspended in 0.1% acetonitrile and analyzed by LC–MS/MS using a nanoRSLC U3000 (Dionex, Thermo Scientific) or a nanoAcquity UPLC (Waters) connected to an LTQ-Orbitrap Velos mass spectrometer or an Orbitrap Fusion Lumos Tribrid mass spectrometer (both Thermo Scientific). The LC gradient was of 60 min for 4%–34% solvent B (Solvent A: 0.1% Formic acid and Solvent B: 80% Acetonitrile/0.1% Formic acid). The survey MS scan was acquired at m/z range 300–1800, and the MS/MS was performed in a data dependent acquisition (DDA) mode. Samples were also analyzed on an Orbitrap Exploris 480 (Thermo Scientific) without FAIMS and with FAIMS at compensation voltage (CV) set at − 50 and − 70 V. For the data independent analysis (DIA), a shorter 15 min LC gradient was used and samples were analyzed using the Exploris 480 in the positive mode from m/z 350–1100 mass range with an isolation window of m/z 15 and 50 scan events (Fig. 1). The Dynamic Exclusion Mode was set to “Auto” where the exclusion time was calculated based on the specified “Expected LC Peak Width” set at 30 s which is multiplied by a factor of 2.5 = 75 s. Auto mass tolerance set at 10 ppm low and 10 ppm high. The settings for the fragmentation type, resolution, mass analyzers and which data are acquired using the Velos, Lumos and Exploris, are described in detail in the supplementary information (Tables S6 and S7).

For peptide identification and label free quantification, the MS/MS data obtained from DDA with and without FAIMS were searched by the SEQUEST search engine using Proteome Discoverer v2.5 (Thermo Scientific). Protein abundances were log transformed and normalized and statistical differences between protein relative abundances were assessed using a t-test as previously discussed [57]. For sequence variant analysis and reporting results based on total spectral counts, the MS/MS data obtained from DDA with FAIMS were exported to .mgf file using Proteome Discoverer v2.5 and submitted to Mascot search engine (v 2.7.0.1) for protein database search using Error tolerant search mode. The Mascot search results were imported as.DAT files into Scaffold v4.6.7 and re-processed to obtain results for Total Spectral Counts. In addition, DDA MS/MS raw files were also analyzed by PEAKS for de novo sequencing. For sequence variants analysis, a customized protein database containing wild type and sequence variant proteins were used for database search while the SwissProt human database was used for all other database searches performed. Other search parameters used were: missed cleavage = 2; carbamidomethylation on C; oxidation on M; deamidation on N and Q; peptide tolerance = 10 ppm; MS/MS tolerance = 0.2 Da for MS/MS spectra acquired in the ion trap analyzer or 0.02 Da for MS/MS spectra acquired in the orbitrap analyzer. Data independent analysis (DIA) data were analyzed using DIA-NN [58].

### Amyloid protein subtyping by MALDI-MSI

FFPE tissue sections at 4 µm thickness were mounted on indium-tin oxide (ITO) conductive glass slides. The FFPE tissue sections were deparaffinized for 1.5 h at 60–65 °C and washed through a series of xylene, 100%, 95%, 70% ethanol and water. Tissue slides in 10 mM Tris buffer were heated to 95–98 °C using a steamer for 30 min. Slides were cooled down, buffer exchanged with water and dried in desiccator overnight [54]. The tissue slides were then spray coated with trypsin solution using the TM Sprayer (HTX Technologies). Trypsin (0.05 µg/µl; in 50 mM ammonium bicarbonate/10% acetonitrile) was sprayed on the tissue slide using the following parameters: nozzle temperature (30 °C), 10 passes, 0.025 mg/ml flow rate, 750 mm/min nozzle velocity, 2 mm track spacing, HH spray pattern, nitrogen gas pressure set at 10, nitrogen gas flow rate (3 L/min), 0 s drying time, 40 mm nozzle height. The total amount of trypsin was 20 µg per slide. The slides were air dried for 5 min and then incubated in a hydrated chamber for overnight trypsin digestion at 37 °C. After digestion, slides were dried for 15 min in the vacuum desiccator and then spray coated with CHCA matrix (α-cyano-hydroxycinnamic acid; 5 mg/ml in 50% AcN/0.2% TFA) using the TM Sprayer (HTX technologies) with the following settings: nozzle temperature (75 °C), 4 passes, 0.1 ml/min flow rate, 1300 mm/min nozzle velocity, 2 mm track spacing, CC spray pattern, nitrogen gas pressure set at 10, nitrogen gas flow rate (3 L/min), 10 s drying time, 40 mm nozzle height. The final matrix density was 0.00077 mg/mm^2^ or 0.8 µg/mm^2^. Peptide spatial mapping was performed by scanning and acquiring spectra m/z range of 700–3500 at 100 μm raster width using the Ultraflextreme MALDI TOF/TOF mass spectrometer (Bruker Daltonics) or 50 μm raster width timsTOF flex mass spectrometer (Bruker Daltonics) equipped with a MALDI-2 laser and Trapped Ion Mobility (TIM). MALDI-TOF data obtained from the Ultraflextreme were processed by FlexImaging software (v 4.0) and Fleximaging (v. 5.0). The timsTOF flex use the SciLs Lab software for data processing to generate amyloid peptide ion maps. For timsTOF fleX MALDI-2 data, several built in functions were used to visualize data, including segmentation. To perform segmentation analysis, a feature list was generated by taking the 10,000 most frequent features across all pixels in the entire m/z range, with the most frequent features being present in the most pixels, regardless of relative intensity. Features are determined upon importation of the data into SCiLS Lab, where the raw data is binned into features based on the default SCiLS binning parameters, and can be thought of as m/z picked peaks. Segmentation was performed by clustering pixels, using the mass spectra of each pixel across the entire m/z range, using only the features found in the feature list mentioned. The clustering was performed using a bisecting k-means algorithm that utilizes correlation distance as a metric, with each pixels spectrum both denoised using the weak denoising setting and normalized against the total ion count of the respective pixel. Further statistical analysis was performed using SCiLS Lab’s built in 10 component principle component analysis using the same 10,000 feature list, normalizing each pixel to the total ion count and scaling the relative intensities of the features to unit variance.

## Results and discussion

### Amyloid protein identification from tissue specimens analyzed by laser capture microdissection and liquid chromatography tandem mass spectrometry (LMD/LC–MS/MS) and data dependent acquisition (DDA) method

Our microdissected and processed tissue specimens were analyzed in DDA mode and identified spectra were filtered for ≥ 95% peptide and ≥ 95% protein probabilities with at least 1 unique peptide and minimum of 3 spectral matches. The amyloid protein identification is based on the proteins list obtained after the protein database search. For example, Figure S1, shows a total number of 342 proteins from tissue specimens collected from Congo red negative controls from bone marrow and heart biopsy versus Congo red positive heart biopsy is reported in a table showing a list of protein names and corresponding total spectral counts for each sample. Transthyretin amyloid protein subtype was identified from the three biological replicates from the Congo red positive heart biopsy sample (ATTR-H-4) together with signature proteins Serum amyloid P component and Apolipoprotein E. The transthyretin amyloid protein was absent in the Congo red negative controls from bone marrow (CRN-B-3) and heart (CRN-H-2). Therefore, these specimens were sub-typed as transthyretin amyloidosis or “ATTR”.

**Fig. 2:**
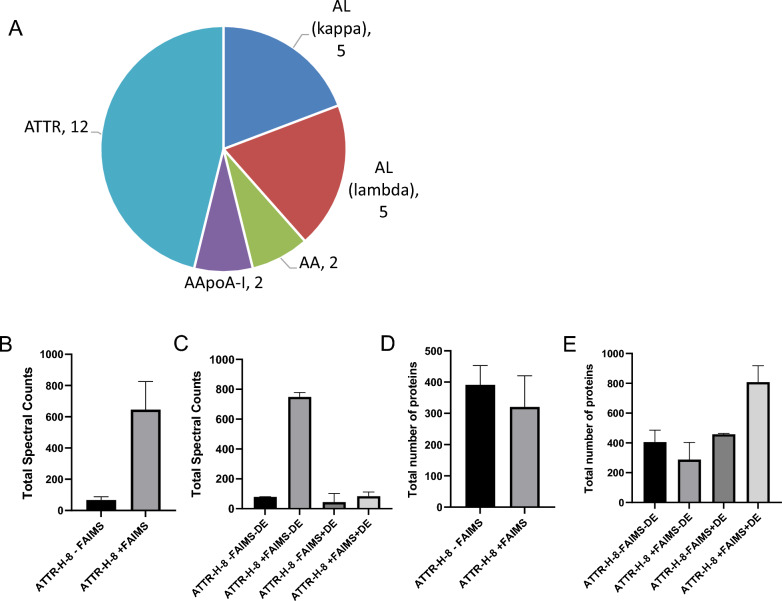
**A** Amyloidosis sub-types from tissue specimens analyzed by LMD/LC–MS/MS. **B** Total spectral counts for transthyretin protein from CR (+) ATTR specimens analyzed by ± FAIMS with no dynamic exclusion (DE). **C** Total number of proteins identified from CR (+) ATTR specimens with and without FAIMS with no dynamic exclusion (DE). **D** Total number of proteins identified with and without FAIMS (± FAIMS) with and without dynamic exclusion **E** Total spectral counts of amyloid proteins with and without FAIMS with and without dynamic exclusion (± DE)

We analyzed 31 tissue specimens, i.e., 5 Congo red negative and 26 Congo red positive with the corresponding number of samples per amyloid protein sub-type identified (Fig. 2A).

Out of 31 specimens analyzed, 46% of the specimens analyzed were sub-typed as transthyretin amyloidosis (ATTR) (Fig. 2A, Table S1, Figure S2A and S2B). Out of the twelve ATTR subtypes, ten specimens were from heart, one from nerve and one from stomach. This indicates that cardiac ATTR is the most prevalent amyloidosis subtype among the specimens we have analyzed. Transthyretin is synthesized in the liver and functions as a transport protein for thyroxine hormone and retinol (Vitamin A). The wild type form (senile ATTR amyloidosis) is observed in older patients over 65 years old. The hereditary form of ATTR has 120 sequence variants of transthyretin [10, 19, 21]. Both the wild type and mutant forms of transthyretin can result in extracellular accumulation of amyloid fibrils that can be localized or systemic resulting in organ failure.

Immunoglobulin light chain amyloidosis (AL) is one of the most common types of amyloidosis. We identified AL-kappa and AL-lambda, each accounting for 19% of the amyloidosis types in our dataset. The organ source varied from lymph node, kidney, heart, intestine, stomach, colon and thigh mass (Fig. 2A, Table S1, Figure S2C and S2D). Our proteomics analysis highlighted that careful differential diagnosis between cardiac AL from cardiac ATTR amyloidosis (wild type and Val122I variants) is possible using our pipeline. This is critical, because ATTR has clonal immunoglobulin abnormality and resembles AL amyloidosis, which may result in misdiagnosis of AL. This could lead to incorrect treatment by unnecessary high-dose chemotherapy or autologous peripheral blood stem cell transplantation, while ATTR requires liver transplantation [21, 59].

Apolipoprotein-A1 amyloidosis (AApoAI) was identified from the lung and colon specimens (Fig. 2A, Table S1). APOA1-associated gastrointestinal and pulmonary amyloidosis have been reported and support our results [60, 61]. Apolipoprotein A-I is synthesized in the liver and small intestine and is the main protein component in high density lipoprotein (HDL) and together they transport cholesterol from peripheral tissues to the liver. Excessive systemic accumulation of misfolded APOA1 fibrils in tissues can result in APOA1 amyloidosis which is rare and can be wild type form or as hereditary variants [16, 62, 63]. There are about 20 variants associated with APOA1 amyloidosis found on hot spots from residues 50–93 and 170–178 [63]. APOA1 is often misdiagnosed or undiagnosed by other methods but the combined Congo red staining and laser capture microdissection with LC–MS/MS has greater sensitivity to enable the identification of the APOA1 amyloid protein.

Two kidney specimens from our cohort were subtyped as AA due to the presence of Serum amyloid A1 protein (Fig. 2A, Table S1). Both Serum Amyloid A1 (SAA1) and A2 (SAA2) isoforms are synthesized in the liver mostly bound to apolipoproteins such as APOA1 or APOE that bind to high density lipoproteins (HDL). SAA is produced in response to pro-inflammatory cytokines such as IL-1, IL-6 and tumor necrosis factor-alpha (TNF-α) [64]. Individuals under prolonged inflammatory conditions due to infectious diseases, hereditary auto-inflammatory diseases, rheumatoid arthritis, granulomatous disease, familial Mediterranean fever malignancies and HIV, have significantly elevated levels of Serum Amyloid A proteins which progress to extracellular deposition of fibrils that cause renal amyloidosis [65]. This is due to the undegraded form of this protein formed from incomplete proteolysis of Serum amyloid A in lysosomes and macrophages that end up bound to proteoglycans and Serum Amyloid P component protein. The kidney is the most commonly affected organ that progress to renal failure under prolonged inflammatory state and if AA is undiagnosed earlier. About 97% of renal amyloidosis is due to glomerular deposition in tubulointerstitium and/or interlobular arteries and arterioles [66].

We confirmed that mass spectrometry has multi-protein detection capability in the analysis of amyloidosis specimens, which is particularly important since amyloidosis is a heterogeneous group of disorders. Having specimens with more than one highly abundant amyloid protein is highly possible and creates ambiguity leading to equivocal results. It is therefore imperative that the test result should contain a full list of all the proteins confidently identified from every biological replicate derived from clinical specimens to help pathologists carefully diagnose the correct amyloid protein subtype(s) (Table S2).

### High-field asymmetric waveform ion mobility spectrometry (FAIMS) boosts sensitivity for low sample input collected from amyloidosis specimens

The use of high-field asymmetric waveform ion mobility spectrometry (FAIMS) has proved to increase sensitivity for ultra-low input samples [67]. By using two compensation voltages at − 50 and − 70 V, we demonstrated that FAIMS significantly improved the sensitivity of our analysis. Specifically, the spectral counts (peptide-spectrum matches) for the transthyretin amyloid protein are ten times higher compared to the same biological replicates analyzed without FAIMS on the same LC–MS/MS setup (Fig. 2B).

We also explored the dynamic exclusion of the acquisition method, i.e. the time interval in DDA acquisition to “ignore” a given precursor to prevent its continuous re-selection. Due to the relatively small number of precursor ions present in our sample(s), we demonstrated that the selection of transthyretin amyloid peptides for fragmentation increased in frequency when the dynamic exclusion was disabled (Fig. 2C). The higher number of spectra for transthyretin peptides improves amyloid protein subtyping, which is the major goal of this pipeline (Fig. 2C). However, this did not result in an increased protein identification, regardless of the use of FAIMS when dynamic exclusion is disabled (Fig. 2D and E). On the other hand, with FAIMS and dynamic exclusion enabled, the number of protein identifications doubled (Fig. 2E) but it drastically lowered the total spectral counts for the transthyretin peptides (Fig. 2C). The increase in the number of protein identification only becomes useful for increasing the depth of the patients’ proteome profile that could be valuable for determining personalized treatment options or a cross comparison of patients’ proteome profiles for biomarker discovery that can be translated as diagnostic markers or potential drug targets other than the amyloid proteins.

### Label free quantitation based on spectral counting versus MS1 precursor intensities

**Fig. 3:**
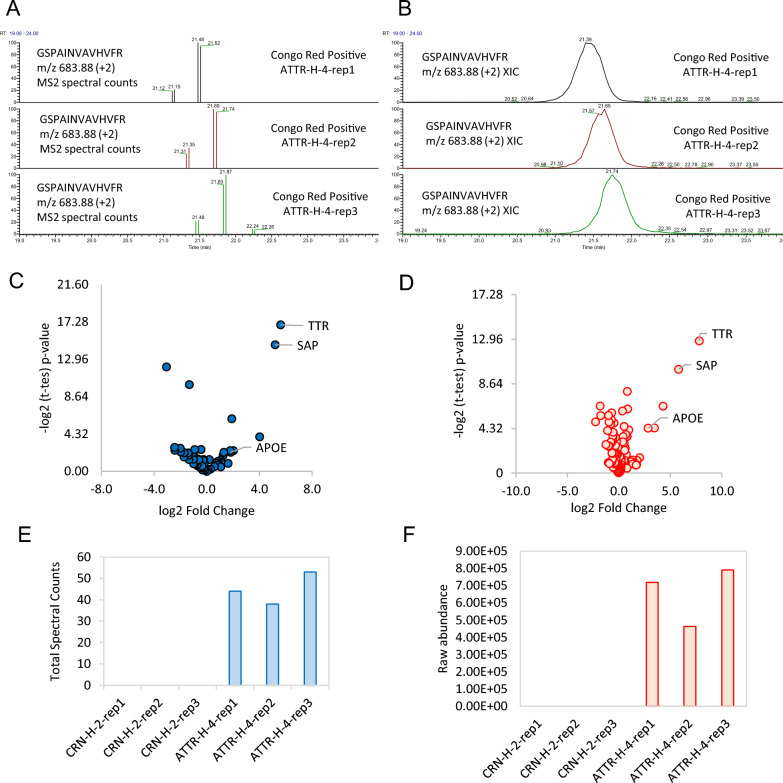
Two label free quantitation methods based on **A** Spectral counts based on peptide-spectral matches at the MS2 level versus **B** Extracted ion chromatogram (XIC) of peptide GSPAINVAVHVFR (m/z 683.88, + 2) at the MS1 level. **C**, **D** Volcano plots of Congo red negative heart biopsy (CRN-H-2) vs Congo red positive heart biopsy (ATTR-H-4) based on **C** total spectral counts versus **D** normalized abundances of all the proteins identified. **E.** Transthyretin total spectral counts versus **F** raw abundances of transthyretin from Congo red negative heart biopsy (CRN-H-2) versus Congo red positive heart biopsy (ATTR-H-4)

Even though proteomics applied to basic science is mostly performing quantification via extracted ion chromatography, amyloid protein identification based on spectral counts has remained the recommended format for reporting test results for ease in clinical interpretation of results by clinicians. Spectral counting is today considered a semi-quantitative approach [68], which is highly robust but with limited linear dynamic range for protein quantification compared to extracted chromatography. We compared these two label free quantification methods using a two-group comparison between a Congo red negative controls such as heart biopsy specimen (CRN-H-2) versus Congo red positive heart biopsy specimen (ATTR-H-4) (Fig. 3A–F). The total spectral counts based on the MS2 level which is the total number of MS/MS or peptide-spectral matches (Fig. 3A) versus MS1 based peak area (or extracted ion chromatogram–XIC) of the precursor ion for peptide, GSPAINVAVHVFR (m/z 683.88, + 2), are shown in Fig. 3A and B. The two tables of raw data from all the proteins identified were both log2 transformed and normalized resulting in two volcano plots that looked similar, with transthyretin (TTR) amyloid protein as the most significantly enriched in the Congo red positive heart biopsy specimens (Fig. 3C and D). Both the total spectral counts and protein raw abundance for transthyretin also showed similar patterns (Fig. 3E and F). Based on our results, the two label free quantitation methods are comparable which may be due to the enrichment of amyloid protein fibrils collected by laser capture microdissection resulting in boosting the abundance of the amyloid protein compared to other proteins in the sample.

### Data dependent acquisition (DDA) versus data independent acquisition (DIA) modes for amyloid protein identification

**Fig. 4:**
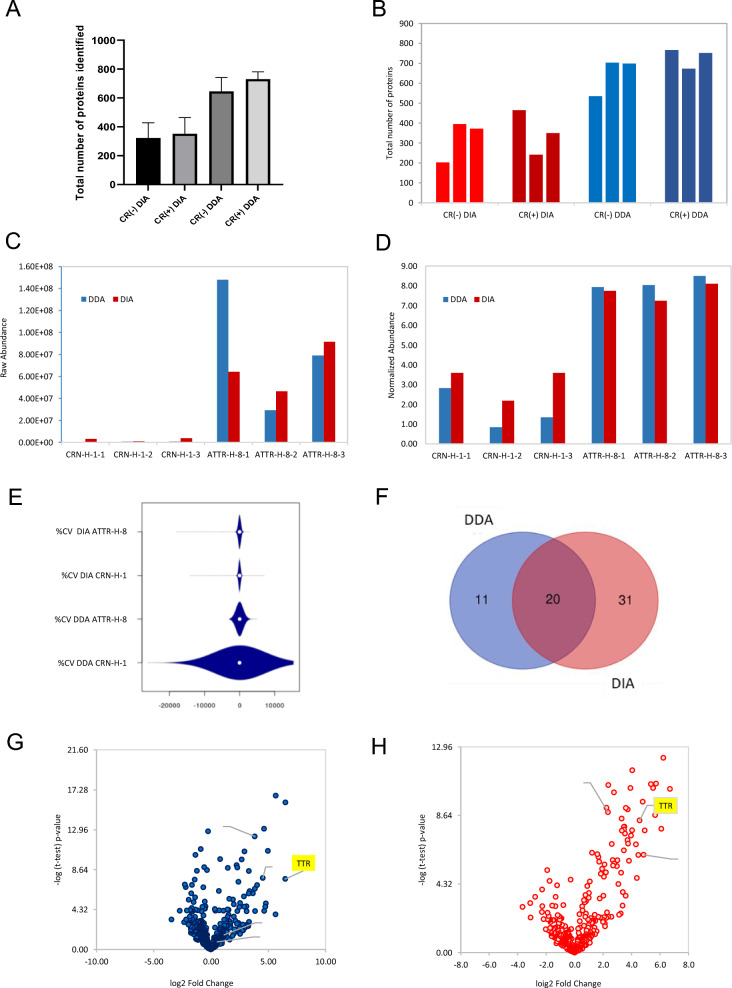
**A** Mean total number of proteins identified from Congo red positive CR (+) and negative CR (−) samples analyzed by DDA versus DIA. **B** Total number of proteins identified from each replicate from each group (shown in Panel **A**). **C** Transthyretin protein raw abundances from CR (+) and CR (−) samples by DDA versus DIA. **D** Normalized abundances of the transthyretin protein from CR (+) and CR (−) specimens by DDA versus DIA. **E** Violin plot of % coefficient of variation from DDA versus DIA analysis of CR (+) versus CR (−) samples. **F** Venn diagram of significant proteins identified from DDA and DIA analysis of CR (+) and CR (−) samples. Volcano plots of proteins identified from CR(+) samples versus CR (−) samples analyzed by: **G** DDA with transthyretin protein as one of the most significantly enriched (p-value = 7.62) among the 31 significant proteins in the CR (+) samples (with p-values > 4.32) versus **H** DIA with transthyretin protein as one of the most significantly enriched (p-value = 8.33) among the 51 significant proteins identified in the CR (+) samples (with p-values > 4.32)

Unlike the DDA method, the data independent acquisition (DIA) method involves dividing the precursor mass range into isolation windows relatively larger than with DDA for MS/MS fragmentation, resulting in mixed MS/MS spectra but with the advantage of selecting all the signals at every duty cycle of the mass spectrometer. Recently, DIA has become more popular for proteomics due to this aspect of not leaving any ion unfragmented, including for the discovery of biomarker candidates from clinical samples derived from plasma [69], cerebrospinal fluid [70], urine [71, 72], tissues [73], cell lysates [74] and in single cell proteomics analysis with low input sample amounts [75]. Here, we explored the applicability of DIA for amyloid protein identification. Three Congo red negative (CR−) samples (CRN-H1-1 to CRN-H1-3) and three Congo red positive (CR+) for transthyretin samples (ATTR-H8-1 to ATTR-H8-3) were run back-to-back using DDA and DIA methods. DIA from a 15-min gradient remarkably replicated the results obtained from DDA from a 60-min gradient when the Congo red negative (CR−) samples (CRN-H1-1 to CRN-H1-3) and three Congo red positive (CR+) for transthyretin samples (ATTR-H8-1 to ATTR-H8-3) samples were compared (Fig. 4A and B). Transthyretin (TTR) was identified and quantified to be ≥ 6 orders of magnitude higher in the Congo red positive samples vs the Congo red negative ones using both DDA and DIA (Fig. 4C and D). Despite DDA having a higher number of total proteins identified compared to DIA, there is higher variability in protein abundances across samples due to more missing values resulting in higher % coefficient of variation (% CV) in DDA compared to DIA (Fig. 4E). The lower % CV in DIA is due to lower stochasticity in signal extraction resulting in an improved reproducibility of the peptide/protein quantification. With the DIA method we assessed 51 significantly regulated proteins (t-test P-value < 0.05) compared to only 31 assessed by DDA. Twenty (20) of these proteins were found to be significant by both methods (Fig. 4F). The low overlap might mislead to the interpretation that the two methods provide different results; in reality, these differences are due to small changes in the p-value, demonstrated by the very similar volcano plots generated using DDA and DIA data (Fig. 4G and H).

### Sequence variants and post-translational modifications (PTMS) analysis by mass spectrometry

The hereditary ATTR variants have over 90 sequence variants [10], some of which are not present in canonical protein sequence databases such as SwissProt. We therefore performed MS/MS spectra identification analysis using Mascot Error Tolerant Search feature from Matrix Science and the De Novo Sequencing from PEAKS to identify sequence variations and new sequences from a customized Amyloidosis protein database (http://amyloidosismutations.com/). For this, we used DDA runs to search only MS/MS spectra from narrow precursor ion isolations. We detected the transthyretin variant V122I/L (or V142Xle) (Figure S3A). The peptide YTIAALLSPYSYSTTAV**V**TNPK containing the V122I/L variant was identified using Mascot Error Tolerant Search from 6 out of 12 ATTR positive specimens. Figure S3A shows that a possible assignment of Val- > Xle (v) with a mass shift of [+ 14.0156] was identified more than once. Mascot site analysis also localized the Val- > Xle to the second valine residue in the peptide at 94.40% versus the first valine residue at 5.60% probabilities (Figure S3B). The MS/MS assignment also confirmed the shift in mass by + 14.0156 on the second valine residue (Figure S3C). In addition, the non-variant or wild type form of the peptide YTIAALLSPYSYSTTAV**V**TNPK was also identified indicative of heterozygous genotype. These results can also be uploaded and re-processed in Scaffold for validation (Fig. S3D). Altogether, the Mascot Error Tolerance search detected not only the wild type transthyretin but also sequence variant form.

Sequence variants analysis can also be performed using PEAKS, a proprietary software with a de novo sequencing feature (https://www.bioinfor.com/peaks-studio/). This means that PEAKS can determine the amino acid sequence from an MS/MS mass spectrum without using a sequence database. PEAKS was unable to identify V122I/L by de novo sequencing, PTM or spider homology search tool, showing complementarity between the two software tools we used. Possibly, PEAKS missed this identification because it converts all isoleucine to leucine in all protein sequences, and so it is possible that PEAKS does not recognize Unimod’s symbol for those containing Isoleucine/Leucine modification as this is denoted by e.g. V- > Xle modification. In fact, PEAKS was able to identify the V122I/L variant peptide when the V- > Leu was specified in the list of variable modifications (Figure S4A). The PTM and spider search tool of PEAKS [76, 77] also assigned the + 14.02 Da mass shift due to methylation on lysine residues (Figure S4B and C). This is due to modification introduced during the formalin fixation of the tissues [23]. This can introduce ambiguity on the peptide RYTIAALLSPYSTTAVVTNPKE for V122I/L or methylation on K. Figure S4D shows an annotated MS/MS spectrum for the peptide YTIAALLSPYSTTAVVTNPKE when the methylation localizes on lysine K(+ 14.02). This match was generated by the PTM search module of PEAKS. However, the C-terminus of the peptide, i.e. VTNKE, does not have sufficient fragments to unambiguously differentiate between V- > 122I/L and a methylated K. Therefore, caution is advised in interpreting such PTM assignment. We utilized the results of this analysis to create our customized Amyloid protein database that includes the known sequence variants (Table S4). This customized Amyloid protein database can also be edited to add potentially new sequence variants.

Apart from sequence variants and the lysine methylation discussed, mass spectrometry is capable of an unbiased detection of a range of post-translational modifications (PTMs) that can be found on the amyloid protein fibrils. It can tell the type and the site of PTMs based on observed mass shifts and fragmentation patterns. By varying and controlling conditions for sample preparations for LC–MS/MS, MALDI-TOF or MALDI with Imaging, PTMs such as nitration, isomerization, racemization, phosphorylation were identified from beta-amyloid proteoforms in Alzheimer’s disease [78] while oxidation, pyroglutamylation, glycosylation were found in Alzheimer’s and AL amyloidosis [78–81]. Conserved disulphide bonds are common in lambda light chain AL fibrils [79, 82] while S-sulfonation (S-Sulfo), S-glycinylcysteinylation (S-CysGly), S-cysteinylation (S-Cys) and S-glutathionylation (S-GSH) on Cys-10 are found in transthyretin ATTR fibrils [83]. We also found some *N*-glycosylation by performing the error tolerant search on Mascot but this modification was not consistently observed in all samples. The presence of mutations in the encoding gene and PTMs may play a role in inducing conformational changes that favour the formation of the amyloid fibrils that are resistant to degradation [78, 79].

However, it is important to note that mass spectrometry can also detect artifact modification introduced during formalin fixation of tissues [78]. In our case, we used Mascot Error Tolerant for unknown PTMs search. In addition to the sequence variants and glycosylation, methylation and formylation on lysine residues were the modifications were commonly observed due to formalin fixation while carbamidomethylation on cysteine and deamidation on asparagine due to the reduction and alkylation steps performed under basic pH condition. Perhaps we only had 31 specimens analysed and some PTMs as described previously by others are patient specific where it was observed in some patients [79, 83] but not in others [79, 82].

### Discovery proteomics: applications beyond the amyloid protein identification pipeline

**Fig. 5:**
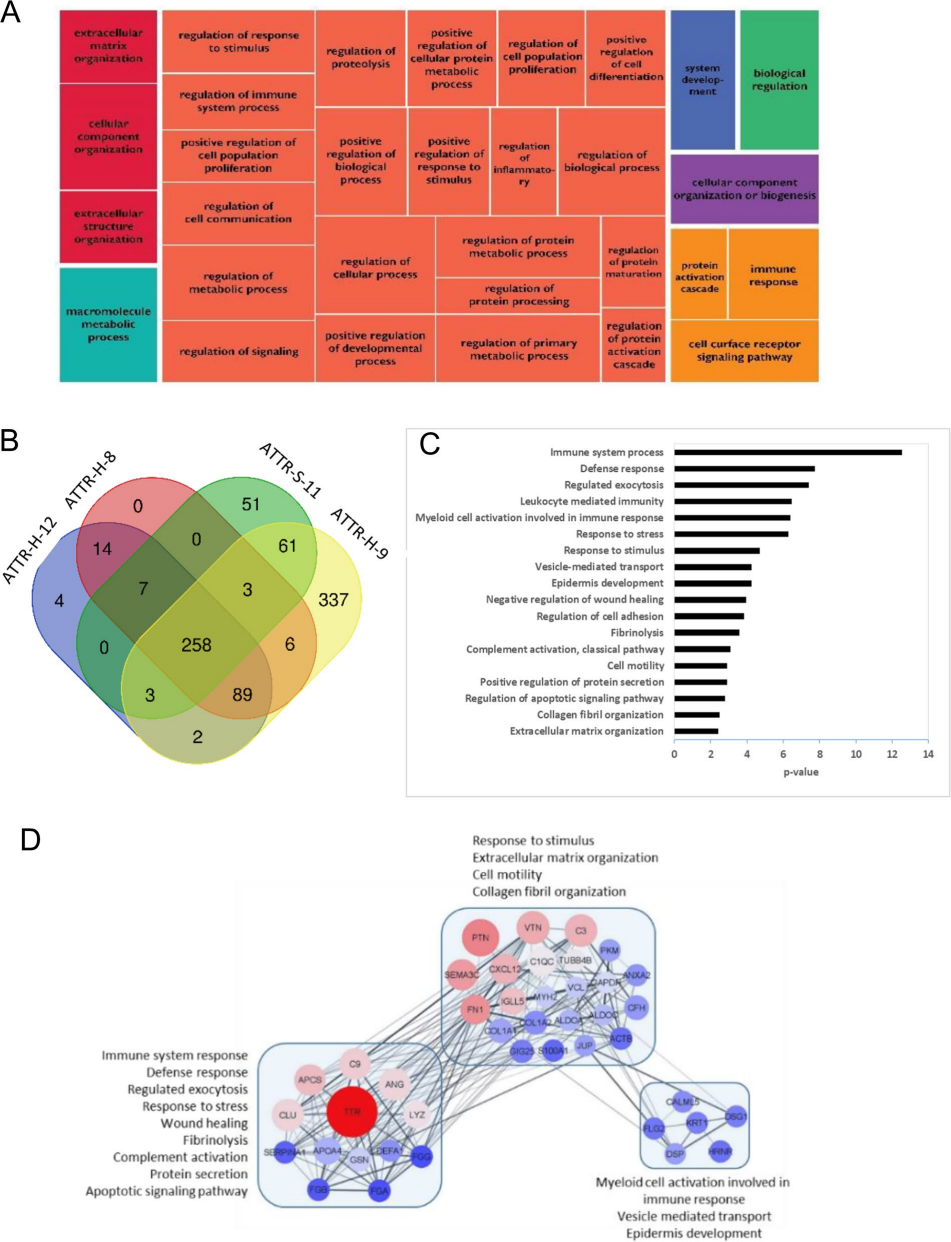
**A** A tree map of biological processes enriched in the Congo red positive specimens** B** Venn diagram of common proteins from heart (ATTR-H-12); heart (ATTR-H-8); stomach (ATTR-S-11); heart (ATTR-H-9); **C** Protein interaction network by STRING and Cytoscape; **D** Gene ontology of the top biological processes

We aim to show in this section some of the tools that can be used for gene ontology analysis that can provide information on biological processes and pathways that pertain to the amyloid protein as well as the other proteins identified around its environment. The analysis of all the proteins detected in the proteomics acquisition, and not just the predicted biomarkers, revealed that extracellular matrix organization, wound healing and fibrinolysis are some of the top biological processes enriched in the amyloidosis proteome (Fig. 5A). These results were obtained by comparing the heart specimen ATTR-H-8 with transthyretin (TTR) amyloidosis versus negative control CRN-H-1. This was expected, being amyloidosis a disorder that involves deposition of amyloid fibrils in the extracellular matrix. A complete list of the identified proteome can be found in Table S3. Transthyretin in its stable form has a tetrameric structure that transports hormones in plasma and has neuroprotective and oxidative-stress-suppressing factor functions. Destabilization of TTR due to aging factors, mutation and changes in post-translational modifications, proteostasis and Ca^+2^ result in cytotoxic transthyretin amyloid fibrils observed and linked in the pathogenesis of senile and hereditary transthyretin amyloidosis (ATTR) [84, 85]. Figure 5B is a Venn diagram that shows the number of proteins that are common among the different amyloid protein sub-types. For transthyretin amyloidosis, angiogenin (ANG) was found to be a protein common in four ATTR specimens from heart and stomach (ATTR-H-8, ATTR-S-11, ATTR-H-9 and ATTR-H-12).

The list of proteins found in the immune system process was re-analyzed by STRING with k-means clustering and found transthyretin and angiogenin to have genetic interaction with other known universal protein markers such as serum amyloid P component, Apolipoprotein A-IV, Clusterin, Complement C9, etc. (Fig. 5C and D) which are pro-inflammatory markers. Aggregated forms of transthyretin that form the amyloid fibrils are linked with immune system response that promotes matrix remodeling [85, 86]. On the other hand, stable transthyretin is a negative marker for inflammation and angiogenin may have anti-inflammatory activity previously reported to have an increase in protein concentrations in serum during the inflammatory response [87]. Angiogenin promotes homeostasis with its dual function of regulating cell growth when it is in the nucleolus and gets translocated to the cytoplasm in response to stress for damage repair and cell survival. However, it had been reported that attenuation of angiogenin anti-stress activity by aggregate deposition of amyloidogenic variant of APOA1 can induce cell death [88]. Overexpression of mutant APOA1 in cells decreased angiogenin expression and altered the cellular location of angiogenin. Under stress conditions, it did not translocate in the cytoplasm but remained in the nucleus promoting rRNA transcription resulting in ribotoxic effects and abrogated its pro-survival function. It is interesting to note that exogenous addition of angiogenin rescued L75P-ApoA-I-induced apoptosis. Perhaps, a similar situation may be happening when transthyretin amyloid protein forms extracellular protein fibrils. Discovering angiogenin as a potential key player in the pathogenesis of amyloidosis, through gene ontology analysis, could be interesting to explore. However, this requires further investigation and is beyond the scope of this paper.

### Amyloidosis subtyping by matrix assisted laser desorption-mass spectrometry imaging (MALDI-MSI)

**Fig. 6:**
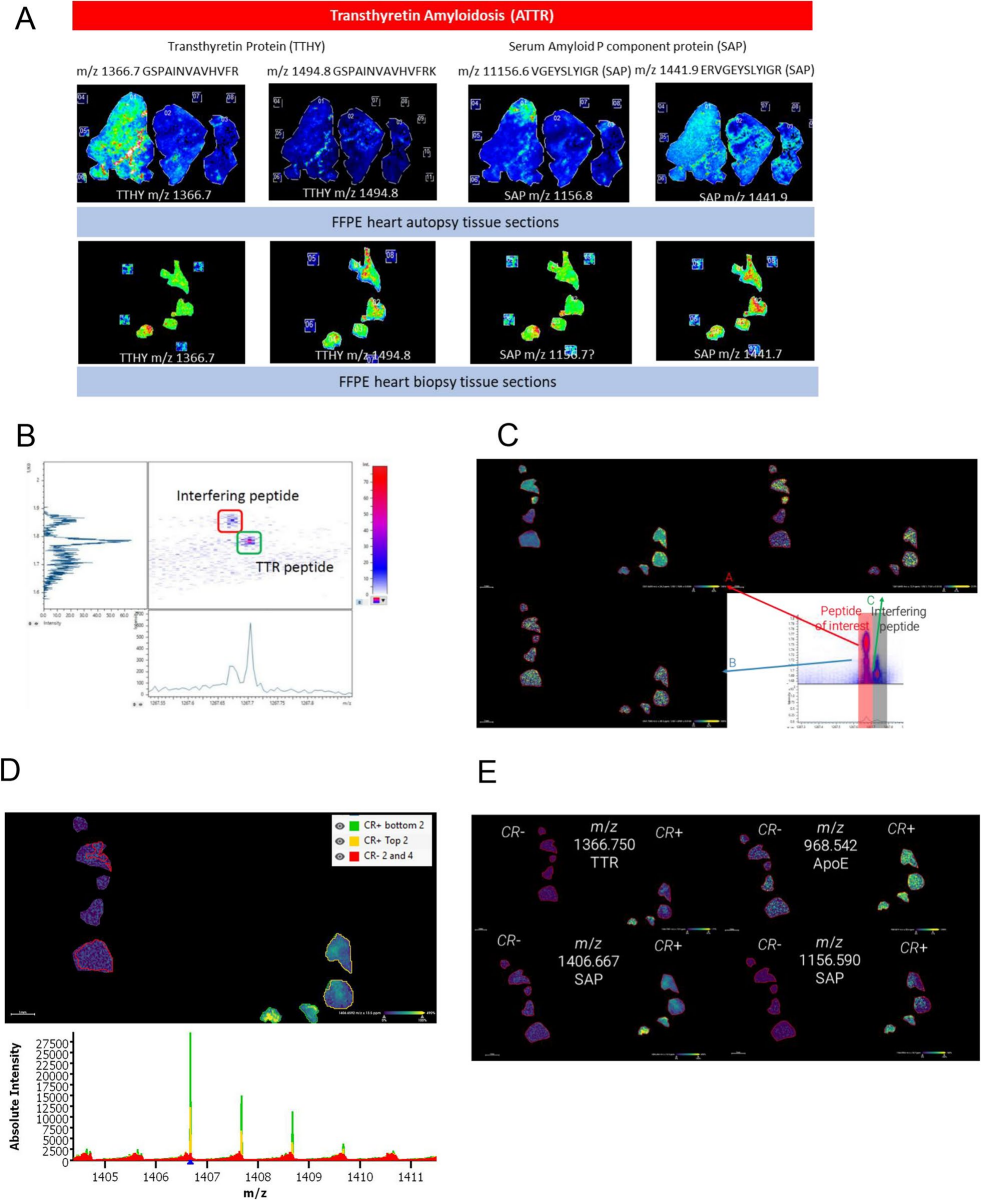
**A** MALDI-MSI analysis of transthyretin peptide (ATTR) GSPAINVAVHVFR (m/z 1366.7) on FFPE heart tissue section from autopsy and biopsy samples using the Ultraflextreme mass spectrometer. Presence of serum amyloid P component peptides VGEYSLYIGR (m/z 1156.6) and ERVGEYSLYIGR (m/z 1441.9) were also mapped on the same tissue sections. **B** Mass mobility resolved interfering ion observed from the Congo red positive peptide extract spiked with the peptide digests of the transthyretin standard**; C** three overlapping peptides observed from complex FFPE heart tissue matrix resolved by TIMS; **D** peptide ion heat maps from transthyretin, serum amyloid P component and Apolipoprotein E; **E** segmentation analysis

MALDI-Mass Spectrometry Imaging (MALDI-MSI) is another promising tool for pathologists, as it enables in situ peptide spatial mapping skipping laser capture microdissection. We subjected to MALDI-MSI, 9 tissue sections from different organs with AL (kappa or lambda) and ATTR amyloidosis subtypes using an Ultraflextreme MALDI-TOF/TOF mass spectrometer (Fig. 6A). The purpose was to show how MALDI-MSI can complement the current technique of laser capture microdissection and tandem mass spectrometry (LMD/LC–MS/MS) for amyloid protein identification. The transthyretin peptide GSPAINVAVHVFR (m/z 1366.7) was the most consistently detected from samples analyzed by LMD/LC–MS/MS and was also detected in MALDI-MSI (Fig. 6A). Because this is an on-tissue trypsin digestion, there is also the probability of missed cleavage and the peptide GSPAINVAVHVFRK (m/z 1494.8) with one missed cleavage was also mapped. Similarly, we could also map the Serum amyloid P component (SAP), a universal protein marker that is associated with the presence of amyloid proteins using the peptides VGEYSLYIGR (m/z 1156.6) and ERVGEYSLYIGR (m/z 1441.9) with 0 and 1 missed cleavage, respectively.

Next, we repeated the analysis with a more recent MALDI-MSI model, the Bruker timsTOF flex. Digested peptides from in-solution trypsin digestion of pure transthyretin standard were used as a positive control and analyzed by MALDI-TOF using the timsTOF flex (Figure S5C). Peptide extracts obtained from on-tissue trypsin digestion of Congo red positive (CR+) and Congo red negative (CR−) FFPE heart biopsy (Figure S5A and S5B) and autopsy specimens (Figure S5D and S5E). Pure transthyretin peptide digests was analysed (Figure S5C) and were spiked in the peptide extracts to test the capability to differentiate isobaric peptides. The timsTOF flex (with mass resolution of 60,000 at m/z 400) was able to detect the well resolved transthyretin peptide GSPAINVAVHVFR (m/z 1366.7) (Figure S6A, S6C and S6D) only from the Congo red positive biopsy, the pure transthyretin standard and autopsy heart specimens while it is absent in the Congo red negative heart specimens (Figure S6B and S6E).

Trapped ion mobility spectrometry (TIMS) separation adds another dimension of identification when combined with MALDI-TOF. Mass and mobility resolved the transthyretin peptide m/z 1267.7 (SYSTTAVVTNPK) from an interfering ion from the Congo red positive (ATTR-H-4) peptide extract spiked with the peptide digests of the transthyretin standard (Fig. 6B) without the tissue matrix.

TIMS is a useful way to screen for candidate peptide markers as overlapping or isobaric peptides are further resolved in the gas phase based on their collision cross sections (Table S5). In the presence of tissue matrix, the acquired MALDI-TOF spectra becomes more complex and there are now three overlapping peptides around m/z 1267.7 resolved by TIMS. Ion maps revealed that the three ions were observed in both Congo red positive and negative heart tissue specimens. This suggests that the peptide ion with m/z 1267.7 is not an ideal marker peptide while the peptide m/z 1366.7 is a potential peptide marker for transthyretin.

Peptide ion heat maps for transthyretin (TTR) peptides (m/z 1366.750) and (m/z 1406.667) were observed to be highly abundant in the Congo red positive (CR+) heart tissue biopsy tissue but not in the Congo red negative (CR−) tissue (Fig. 6B). In addition, peptides from universal markers, serum amyloid P component (SAP) (m/z 1156.590) and Apolipoprotein E (APOE) (m/z 968.542) co-localize with amyloid proteins in the CR+ tissue (Fig. 6C). Segmentation analysis (Fig. 6D and 6E) clustered statistically similar regions and the extracted average mass are used to distinguish between CR- and CR+ tissue biopsies which is further illustrated by the principal component analysis (Fig. S7) showing how different the CR+ from the CR− biopsy samples are from each other. Altogether, we demonstrated that MALDI-MSI is a suitable alternative to LMD/LC–MS/MS for quantifying the main biomarkers in Amyloidosis.

### Cost analysis for amyloidosis sub-typing by LMD/LC–MS/MS versus MALDI-MSI

Tables 1, S8 and S9 provide a comparison and breakdown of the cost per sample for the two techniques. The materials used in LMD/LC–MS/MS costs 2.3 times more than MALDI-MSI. Each method required a special type of slide such as PEN membrane slide for LMD/LC–MS/MS versus ITO slide for MALDI-MSI. Both types of slides are commercially available and can be easily purchased. While both methods have deparaffinization steps, the extra step of Congo red staining is an added cost to the materials and sample preparation time for the LMD/LC–MS/MS method. Both methods required the heat induced epitope retrieval for the de-crosslinking step. However, the LMD/LC–MS/MS has an extra step of sonication for efficient protein extraction. While it is a time-saver, it could be an added equipment and maintenance cost (~ $100K) and by contrast, the MALDI-MSI does not require the additional sonication step. The laser capture microdissection step is an additional time consuming step as this can take up to 1 h or more for collecting Congo red stained amyloid fibrils. The reduction, alkylation and overnight trypsin digestion times take about 16–17 h for the LMD/LC–MS/MS. On the other hand, there are no reduction/alkylation steps in MALDI-MSI but instead requires on-tissue trypsin spraying and overnight incubation also for 16–17 h. Prior to mass spectrometry analysis, the LMD/LC–MS/MS samples require the desalting step while MALDI-MSI requires matrix spraying. Our standard acquisition times are 75 min for LC–MS/MS and 90 min for MALDI-MSI. However, the acquisition times for LC–MS/MS can be shortened to 15 min gradient if DIA approach can be implemented while acquisition times for MALDI-MSI can vary and depend on the spatial resolution and the size of the tissue. In our case, the smaller size biopsy samples (~ 3–5 mm2) took 1.5 h per sample while the bigger size autopsy samples (~ 10–15 mm2) took up to ~ 12 h at 50 µm spatial resolution. About 4 h of data analysis time is usually sufficient for both LMD/LC–MS/MS and MALDI-MSI. Taken together, the total cost of $624 per sample for the LMD/LC–MS/MS is just $30 more than MALDI-MSI ($594) because the price of the instruments were factored in the calculation of the cost of instrument time (Table S8 and S9). Finally, the MALDI-MSI has a shorter turnaround time of 3.3 days versus 4.2 days for LMD/LC–MS/MS which could be an important advantage to consider. Both techniques are amenable to automation if robotics like liquid handlers and automated tissue preparation systems are available. However, this will be added equipment costs to purchase and maintain.

**Table 1:** Cost comparison of amyloidosis subtyping analysis by laser capture microdissection-liquid chromatography tandem mass spectrometry (LMD/LC–MS/MS) versus MALDI mass spectrometry imaging with ion mobility (MALDI-MSI)

Per sample	LMD/LC-MSMS	MALDI-MSI
Materials ($)	73	32
Sample preparation time hands on (h)	3.5	3
Incubation time (h)	25	18.1
Instrument analysis time (h)	1.3	1.5
Data analysis time (h)	4	4
Total analysis time (h)	33.8	26.6
Total analysis time (days based on 8 h work/day)	4.2	3.3
Cost per sample ($)	624	594

## Conclusions

In this work, we demonstrated that the amyloidosis assay validated as a laboratory developed test (LDT) by pathology laboratories is a robust assay for clinical testing, but innovations in mass spectrometry offer opportunity for improved sensitivity, accuracy and alternative workflows. We demonstrated that for data dependent acquisition (DDA) without dynamic exclusion favored a boost in the total spectral counts (TSC) or number of peptide spectrum matches (PSM) of the amyloid protein subtype but resulted in lower total number of proteins identified. On the other hand, DDA with dynamic exclusion increased the total number of proteins identified. Despite the decrease in total TSC or PSMs, the amyloid protein subtype was still identified. DDA with dynamic exclusion resulted in an increased depth of protein coverage by identifying greater number of proteins compared to DDA without dynamic exclusion and this could be very useful in discovery proteomics. We showed that data independent acquisition (DIA) offers a potentially higher accuracy in reproducible quantification and higher throughput due to shorter gradients compared to DDA. In addition to this, the use of FAIMS is greatly beneficial for low protein input from samples obtained from methods such as laser capture microdissection. Further improvements in FAIMS acquisition could be performed by optimizing the compensation voltages.

MALDI mass spectrometry imaging is another promising method to develop that could complement the existing laser capture microdissection/LC–MS/MS method. Direct on tissue trypsin digestion can potentially shorten processing times. On-tissue peptide spatial mapping can provide additional spatial information which could complement the spatial information provided by Congo red staining which is confined to the detection of amyloid protein fibrils. We have shown the transthyretin peptide spatial mapping from ATTR amyloidosis specimens by MALDI-MSI. The application of MALDI in diagnostics specifically for microbial identification using the FDA approved Biotyper serves as a successful model and a good motivation to explore further the potential applications of MALDI mass spectrometry imaging for amyloidosis assay and potentially other clinical assays utilizing tissue specimens.

We demonstrated the use of mass spectrometry for de novo sequencing can potentially detect sequence variants which can perhaps complement the gene sequencing analysis. We have also shown that mass spectrometry is also capable of detecting both post-translational or artefact modification on the amyloid proteins analyzed.

The comparison on the cost of amyloidosis sub-typing on MALDI versus LMD/LC–MS/MS will hopefully provide some idea in terms of costs of materials, instrument time and turnaround time for laboratories that are interested in performing proteomics based testing of clinical samples. Finally, all combined, this paper summarizes a series of potential improvements to enhance an individual’s clinical profile that can be used for the development of personalized medicine or drug therapeutics for amyloidosis.

### Supplementary Information

Supplementary Material 1. ** Fig S1.** Table of proteins identified using Scaffold software. Transthyretin amyloid protein was identified marked with yellow star. Amyloid associated proteins such as Serum amyloid P component and Apolipoprotein E are also marked with yellow star. The total spectral counts for each sample are shown as the numbers shaded in green. Fig. 2 A. Amyloidosis sub-types from tissue specimens analyzed by LMD/LC–MS/MS. **B.** Total spectral counts for transthyretin protein from CR (+) ATTR-H-8 specimens with and without FAIMS (± FAIMS) with no dynamic exclusion (−DE) **C.** Total spectral counts for transthyretin protein from CR (+) ATTR-H-8 specimens with and without FAIMS (± FAIMS) with and without dynamic exclusion (± DE). **D.** Total number of proteins identified from CR (+) ATTR-H-8 specimens with and without FAIMS (± FAIMS) with no dynamic exclusion (−DE). **E.** Total number of proteins identified from CR (+) ATTR-H-8 specimens with and without FAIMS (± FAIMS) with and without dynamic exclusion (± DE). All the MS data acquired with or without FAIMS (± FAIMS) and with or without dynamic exclusion (± DE) were done on the Exploris 480 Orbitrap mass spectrometer. **Figure S3 A.** Mascot search result using error tolerant search for the transthyretin peptide YTIAALLSPYSYSTTAV(V + 14)TNPK with Val- > Xle variant a + 14.0156 mass shift; **B.** Mascot search results with matches to query with site analysis between Val17 and Val18; **C.** MS/MS fragmentation for confirmation of Val- > Xle substitution on Val18; **D.** Mascot search result re-processed in Scaffold with MS/MS fragmentation and assignments from wild type versus sequent variant transthyretin peptide. **Figure S4A.** PEAKS PTM and SPIDER assignment of V122I/L by V- > Leu (Xle) with + 14.02 mass shift on peptide RYTIAALLSPYSTTAV**V**TNPKE; **S5B.** Methylation on peptide RYTIAALLSPYSTTAVVTNP**K**E; **S5C.** both V- > Leu (Xle) and methylation on RYTIAALLSPYSTTAV**V**TNP**K**E. **Figure S5.** On-tissue trypsin digestion of FFPE heart tissue from **A.** Congo red positive (CR+) and **B.** Congo red negative (CR−) biopsy specimen; **C.** Congo red positive (CR+) and **D.** Congo red negative (CR−) biopsy specimen; **E.** in-solution trypsin digestion of a transthyretin protein standard. **Figure S6.** Peptide GSPAINVAVHVFR (m/z 1366.7) as potential biomarker for transthyretin observed only in **A.** Congo red positive heart biopsy (ATTR-H-4), **C.** transthyretin protein standard (TTR-Std-2) and **D.** Congo red positive heart autopsy (ATTR-H-9) but not in the **B.** Congo red negative heart biopsy (CRN-H-2) and **E.** Congo red negative heart autopsy specimen (CRN-H-1). **Fig. S7.** Principal component analysis of Congo red negative biopsy specimen (CRN-H-2) versus Congo red positive specimen (ATTR-H-4)

Supplementary Material 2.** Table S1—**Amyloidosis sub-types from tissue specimens from different organs analyzed by combined laser capture microdissection and liquid chromatography tandem mass spectrometry (LC-MS/MS).** Table S5**—Collision cross section (CCS) values from isobaric peptides observed from CR (+) and CR (−) tissues analyzed and resolved by MALDI-ultrahigh resolution and TIMS. **Table S6**—List of amyloidosis samples and the different platforms used for data acquisition. **Table S7**—Mass spectrometer parameters and settings for the Velos, Lumos and Exploris. **Table S8—**Cost comparison of amyloidosis sub-typing. **Table S9**—Cost analysis amyloidosis subtyping by MALDI-MSI.

Supplementary Material 3.** Table S2**—All worksheets of all specimens analyzed into one Excel file.

Supplementary Material 4 (XLSX 396 KB)** Table S3**—Complete list of all proteins (discovery proteomics).

Supplementary Material 5. **Table S4**—Customised amyloidosis database FASTA file.

## Data Availability

Data are all provided in the Supplementary Tables.
