# Supplementary material for: Effect of dynamic exclusion and the use of FAIMS, DIA and MALDI-mass spectrometry imaging with ion mobility on amyloid protein identification
Source: Clin Proteomics. 2024 Jul 3;21:47. doi: 10.1186/s12014-024-09500-w (PMC11223398; doi:10.1186/s12014-024-09500-w)
Supplement: Supplementary file 1 — Supplementary Material 1. Fig S1. Table of proteins identified using Scaffold software. Transthyretin amyloid protein was identified marked with yellow star. Amyloid associated proteins such as Serum amyloid P component and Apolipoprotein E are also marked with yellow star. The total spectral counts for each sample are shown as the numbers shaded in green. Fig. 2 A. Amyloidosis sub-types from tissue specimens analyzed by LMD/LC–MS/MS. B. Total spectral counts for transthyretin protein from CR (+) ATTR-H-8 specimens with and without FAIMS (± FAIMS) with no dynamic exclusion (−DE) C. Total spectral counts for transthyretin protein from CR (+) ATTR-H-8 specimens with and without FAIMS (± FAIMS) with and without dynamic exclusion (± DE). D. Total number of proteins identified from CR (+) ATTR-H-8 specimens with and without FAIMS (± FAIMS) with no dynamic exclusion (−DE). E. Total number of proteins identified from CR (+) ATTR-H-8 specimens with and without FAIMS (± FAIMS) with and without dynamic exclusion (± DE). All the MS data acquired with or without FAIMS (± FAIMS) and with or without dynamic exclusion (± DE) were done on the Exploris 480 Orbitrap mass spectrometer. Figure S3 A. Mascot search result using error tolerant search for the transthyretin peptide YTIAALLSPYSYSTTAV(V + 14)TNPK with Val- > Xle variant a + 14.0156 mass shift; B. Mascot search results with matches to query with site analysis between Val17 and Val18; C. MS/MS fragmentation for confirmation of Val- > Xle substitution on Val18; D. Mascot search result re-processed in Scaffold with MS/MS fragmentation and assignments from wild type versus sequent variant transthyretin peptide. Figure S4A. PEAKS PTM and SPIDER assignment of V122I/L by V- > Leu (Xle) with + 14.02 mass shift on peptide RYTIAALLSPYSTTAVVTNPKE; S5B. Methylation on peptide RYTIAALLSPYSTTAVVTNPKE; S5C. both V- > Leu (Xle) and methylation on RYTIAALLSPYSTTAVVTNPKE. Figure S5. On-tissue trypsin digestion of FFPE heart tissue from A. Congo [file 12014_2024_9500_MOESM1_ESM.pdf]

Supplementary Figures

Figure S1

A

Probability Legend:
- over 95%
- 80% to 94%
- 50% to 79%
- 20% to 49%
- 0% to 19%

#	Visible?	Starred?	MS/MS View: 342 Proteins in 302 Clusters With 4 Decoys	Accession Number	Molecular Weight	Protein Grouping Ambiguity	CRN-B-3	ATTR-H-4-rep1	ATTR-H-4-rep2	ATTR-H-4-rep3	CRN-H-2-rep1	CRN-H-2-rep2	
1	☑	★	sp|P02766|TTHY_HUMAN Transthyretin OS=Homo sapiens GN=TTR PE...	sp|P02766|TTHY_HUMAN	16 kDa			44	38	53			
2	☑	★	sp|P02743|SAMP_HUMAN Serum amyloid P-component OS=Homo sapi...	sp|P02743|SAMP_HUMAN	25 kDa			35	23	38			
3	☑	★	sp|P01857|IGHG1_HUMAN Ig gamma-1 chain C region OS=Homo sapie...	sp|P01857|IGHG1_HUMAN	36 kDa			9					2
4	☑	★	sp|P02649|APOE_HUMAN Apolipoprotein E OS=Homo sapiens GN=APO...	sp|P02649|APOE_HUMAN	36 kDa			6		8			
5	☑		Cluster of sp|P12883|MYH7_HUMAN Myosin-7 OS=Homo sapiens GN=...	sp|P12883|MYH7_HUMAN	223 k...	★	299	332	332	286	442	357	290
6	☑		Cluster of sp|P68032|ACTC_HUMAN Actin, alpha cardiac muscle 1 OS=...	sp|P68032|ACTC_HUMA...	42 kDa	★	96	99	102	84	128	107	91
7	☑		Cluster of sp|P17661|DESM_HUMAN Desmin OS=Homo sapiens GN=D...	sp|P17661|DESM_HUMAN	54 kDa	★	18	74	73	54	61	69	47
8	☑		Cluster of sp|P35609|ACTN2_HUMAN Alpha-actinin-2 OS=Homo sapie...	sp|P35609|ACTN2_HUM...	104 k...	★	20	56	56	43	78	54	36
9	☑	☆	sp|P25705|ATPA_HUMAN ATP synthase subunit alpha, mitochondrial O...	sp|P25705|ATPA_HUMAN	60 kDa		4	24	41	34	41	37	22
1	☑	☆	sp|P06576|ATPB_HUMAN ATP synthase subunit beta, mitochondrial OS...	sp|P06576|ATPB_HUMAN	57 kDa	★		41	48	42	61	34	30
1	☑	☆	sp|P08590|MYL3_HUMAN Myosin light chain 3 OS=Homo sapiens GN=...	sp|P08590|MYL3_HUMAN	22 kDa	★	12	34	39	30	58	48	35
1	☑		Cluster of sp|P68871|HBB_HUMAN Hemoglobin subunit beta OS=Hom...	sp|P68871|HBB_HUMAN	16 kDa	★	2	39	21	34	58	13	41
1	☑	☆	sp|P00560|PGK_YEAST Phosphoglycerate kinase OS=Saccharomyces ce...	sp|P00560|PGK_YEAST	45 kDa								

Fig S1. Table of proteins identified using Scaffold software. Transthyretin amyloid protein was identified marked with yellow star. Amyloid associated proteins such as Serum amyloid P component and Apolipoprotein E are also marked with yellow star. The total spectral counts for each sample are shown as the numbers shaded in green.

Figure S2

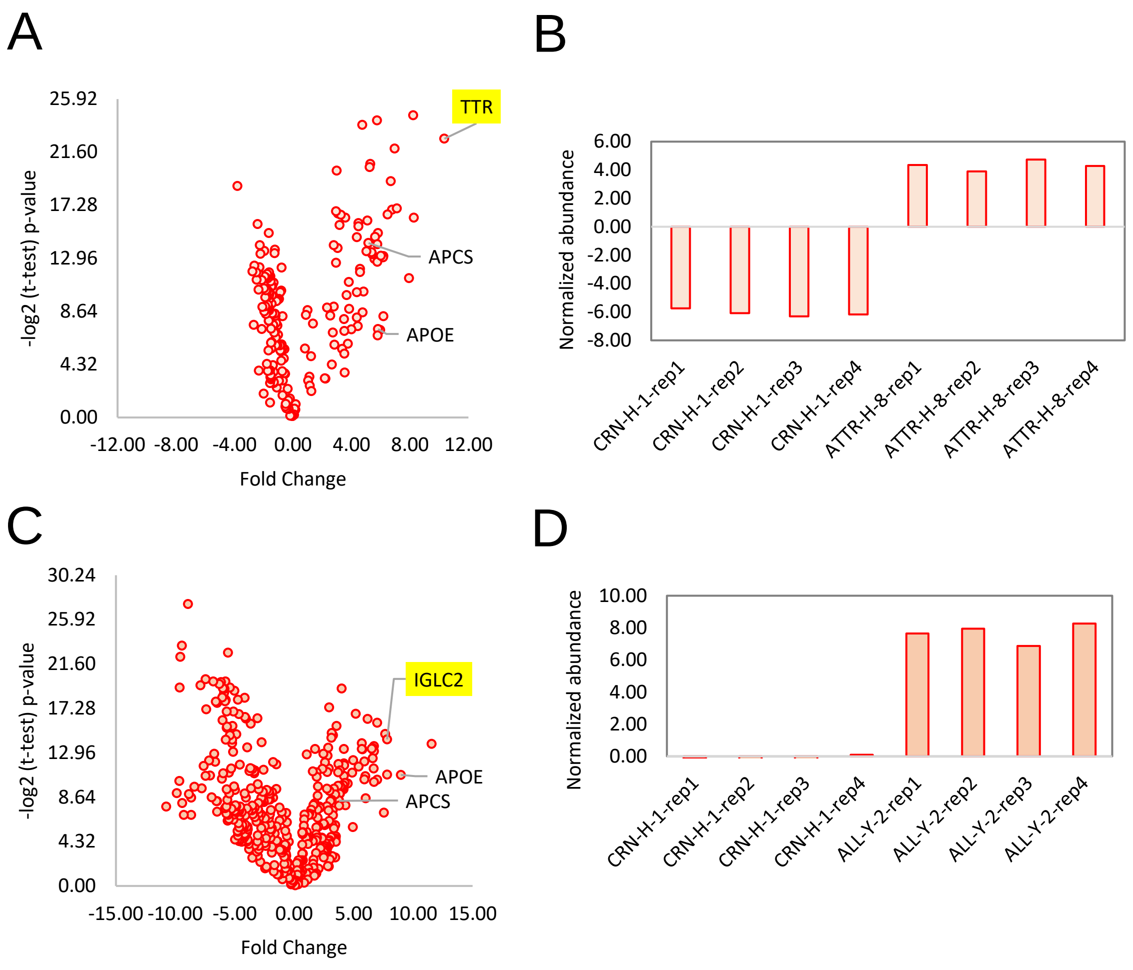

Figure S2. A. Volcano plot of Congo red negative heart autopsy specimen CRN-H-1 versus Congo red positive heart autopsy specimen for transthyretin amyloidosis (ATTR-H-8)**; B.** Transthyretin normalized abundance in CRN-H-1 versus ATTR-H-8; **C.** Volcano plot of Congo red negative heart CRN-H-1 versus Congo red positive lymph node for AL amyloidosis (ALL-Y-2); **D.** Immunoglobulin lambda light chain in CRN-H-1 “H-A-009” vs Congo red positive lymph node (ALL-Y-2).

Figure S3

A

▼8 P02766 439 Transthyretin OS=Homo sapiens OX=9606 GN=TTR PE=1 SV=1

		Score	Mass	Matches	Sequences	
8.1	P02766	439	15991	47 (14)	22 (7)	Transthyretin OS=Homo sapiens OX=9606 GN=TTR PE=1 SV=1

▼*47 peptide matches (43 non-duplicate, 4 duplicate)*

☑ Auto-fit to window

Query	Dupes	Observed	Mr (expt)	Mr (calc)	ppm	M	Score	Expect	Rank	U	Peptide
5531		714.8895	1427.7645	1427.7629	1.14	1	56		1	U	K.CPLMVKVLDAVR.G + [+27.9949 at K6]
5708		722.8855	1443.7564	1443.7578	-0.93	1	71		1	U	K.CPLMVKVLDAVR.G + Oxidation (M); [+27.9949 at K6]
5711		482.2718	1443.7935	1443.7942	-0.50	1	23		1	U	K.CPLMVKVLDAVR.G + Oxidation (M); [+28.0313 at K6]
6074		498.9559	1493.8459	1493.8467	-0.54	1	33	0.002	1	U	R.GSPAINVAVHVFRK.A
6253		761.8621	1521.7096	1521.7100	-0.26	1	73	1.5e-07	1	U	R.KAADDTWEPFASGK.T
6473		775.8599	1549.7052	1549.7161	-7.06	0	33		1	U	K.AADDTWEPFASGK.T + [+156.1011 at N-term]
8336		589.2802	1764.8186	1764.8180	0.37	0	35		1	U	F.HEHAEVVFTANDSGPR.R
9034		928.9662	1855.9179	1855.9203	-1.29	1	58		1	U	L.LSPYSYSTTAVVTNPKE.-
10293		1021.0257	2040.0368	2040.0415	-2.29	1	58		1	U	A.ALLSPYSYSTTAVVTNPKE.-
10294		681.0203	2040.0391	2040.0415	-1.17	1	32		1	U	A.ALLSPYSYSTTAVVTNPKE.-
10825		713.3475	2137.0208	2137.0215	-0.35	0	51		1	U	E.SGELHGLTTEEEFVEGIYK.V
11493		567.5263	2266.0761	2266.0767	-0.25	0	35		1	U	L.GISPFHEHAEVVFTANDSGPR.R
11629		1146.1001	2290.1856	2290.1845	0.50	1	23		1	U	R.RYTIAALLSPYSYSTTAVVTN.P
12076		792.0900	2373.2481	2373.2468	0.55	0	56		1	U	R.YTIAALLSPYSYSTTAVVTNPK.E + [+14.0156 at V18]
12284		810.3957	2428.1652	2428.1659	-0.28	0	40		1	U	K.ALGISPFHEHAEVVFTANDSGPR.R + [-22.0320 at H1[illegible]
12363	1	817.7394	2450.1965	2450.1979	-0.56	0	69	3.1e-07	1	U	K.ALGISPFHEHAEVVFTANDSGPR.R
12364		613.5567	2450.1977	2450.1979	-0.073	0	38	0.00029	1	U	K.ALGISPFHEHAEVVFTANDSGPR.R
12367		818.0671	2451.1794	2451.1819	-1.01	0	26	0.0039	1	U	K.ALGISPFHEHAEVVFTANDSGPR.R + Deamidated (NQ)
12368		613.8026	2451.1813	2451.1819	-0.22	0	27	0.0027	1	U	K.ALGISPFHEHAEVVFTANDSGPR.R + Deamidated (NQ)
12538		627.8125	2507.2209	2507.2193	0.62	0	35		1	U	K.ALGISPFHEHAEVVFTANDSGPR.R + [+57.0215 at H10]
12564		839.4509	2515.3308	2515.3322	-0.57	1	68	4.5e-07	1	U	R.RYTIAALLSPYSYSTTAVVTNPK.E
12592		844.1232	2529.3477	2529.3479	-0.074	1	43		1	U	R.RYTIAALLSPYSYSTTAVVTNPK.E + [+14.0156 at V19]
12901		882.4653	2644.3740	2644.3748	-0.31	1	73		1	U	R.YTIAALLSPYSYSTTAVVTNPKE.- + [+156.1011 at N-term]
12911		883.1370	2646.3891	2646.3790	3.78	2	53		1	U	R.RYTIAALLSPYSYSTTAVVTNPKE.- + [+2.0042 at Y13]
12935		887.1372	2658.3898	2658.3905	-0.25	1	46		1	U	R.RYTIAALLSPYSYSTTAVVTNPK.E + [+143.0582 at C-term K]

Possible assignments:
Val->Xle (V) [+14.0156]

B

All matches to this query

Score	Mr(calc)	Delta	Sequence	Site Analysis
56.0	2373.2468	0.0013	YTIAALLSPYSYSTTAVVTNPK	Val->Xle V18 94.40%
43.9	2373.2468	0.0013	YTIAALLSPYSYSTTAVVTNPK	
43.8	2373.2468	0.0013	YTIAALLSPYSYSTTAVVTNPK	Val->Xle V17 5.60%
33.0	2373.2468	0.0013	YTIAALLSPYSYSTTAVVTNPK	
33.0	2373.2468	0.0013	YTIAALLSPYSYSTTAVVTNPK	
33.0	2373.2468	0.0013	YTIAALLSPYSYSTTAVVTNPK	
31.2	2373.2468	0.0013	YTIAALLSPYSYSTTAVVTNPK	
31.2	2373.2468	0.0013	YTIAALLSPYSYSTTAVVTNPK	
22.3	2373.2468	0.0013	YTIAALLSPYSYSTTAVVTNPK	

C

MATRIX SCIENCE **Mascot Search Results**

Peptide View

MS/MS Fragmentation of YTIAALLSPYSYSTTAVVTNPK

Found in P02766 in SwissProt_AC, Transthyretin OS=Homo sapiens OX=9606 GN=TTR PE=1 SV=1

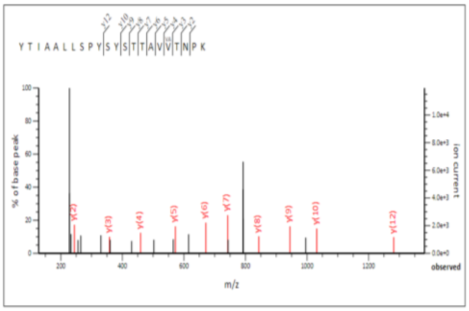

Figure S3A. Mascot search result using error tolerant search for the transthyretin peptide YTIAALLSPYSYSTTAV(V+14)TNPK with Val->Xle variant a +14.0156 mass shift; **B.** Mascot search results with matches to query with site analysis between Val17 and Val18; **C.** MS/MS fragmentation for confirmation of Val->Xle substitution on Val18; **D.** Mascot search result re-processed in Scaffold with MS/MS fragmentation and assignments from wild type versus sequent variant transthyretin peptide.

D

- **Error tolerant search + manual verification of MS/MS spectra**
- **Transthyretin mutation ^122^V to I/L detected from heart tissue**

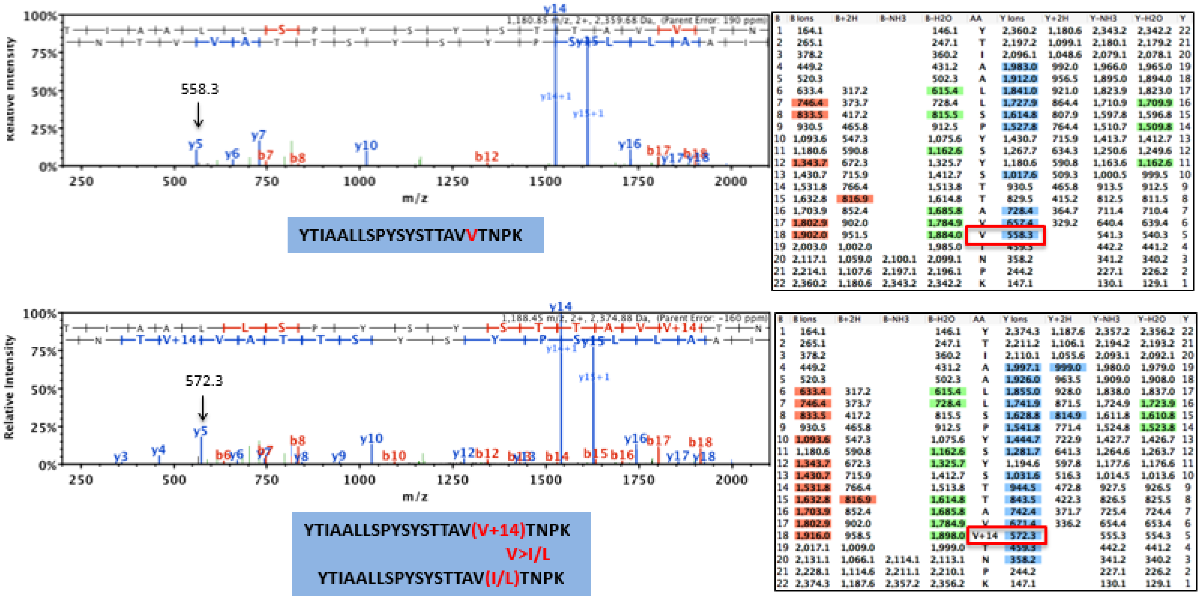

Figure S4

A

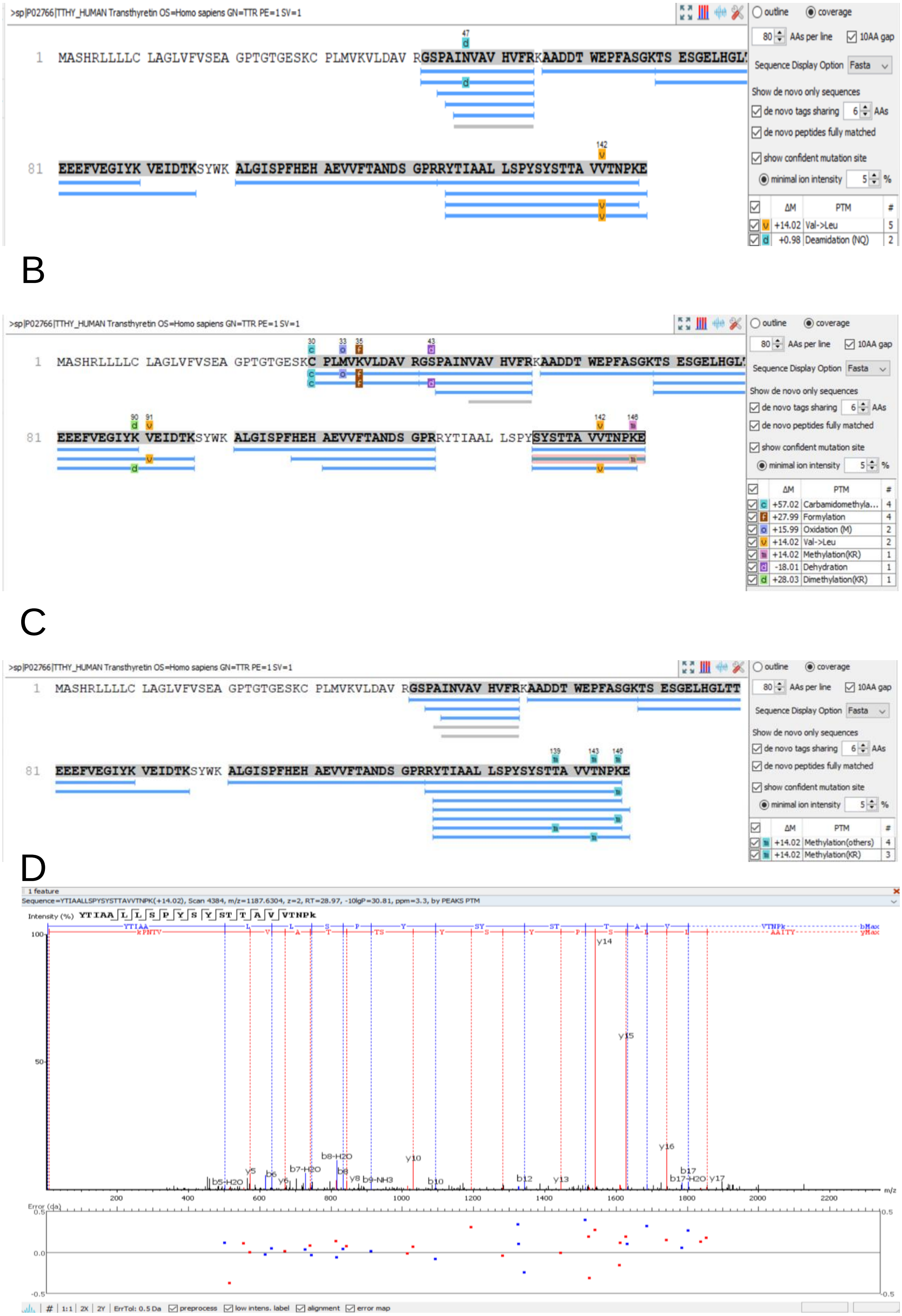

Figure S4A. PEAKS PTM and SPIDER assignment of V122I/L by V->Leu (Xle) with +14.02 mass shift on peptide RYTIAALLSPYSTTAV**V**TNPKE; **SB.** both V->Leu (Xle) and methylation on RYTIAALLSPYSTTAV**V**TNP**K**E. **S4C.** Methylation on peptide RYTIAALLSPYSTTAVVTNP**K**E; **S4D.** MS/MS assignment for RYTIAALLSPYSTTAVVTNP**K(+14)**E (methyl on K).

Figure S5

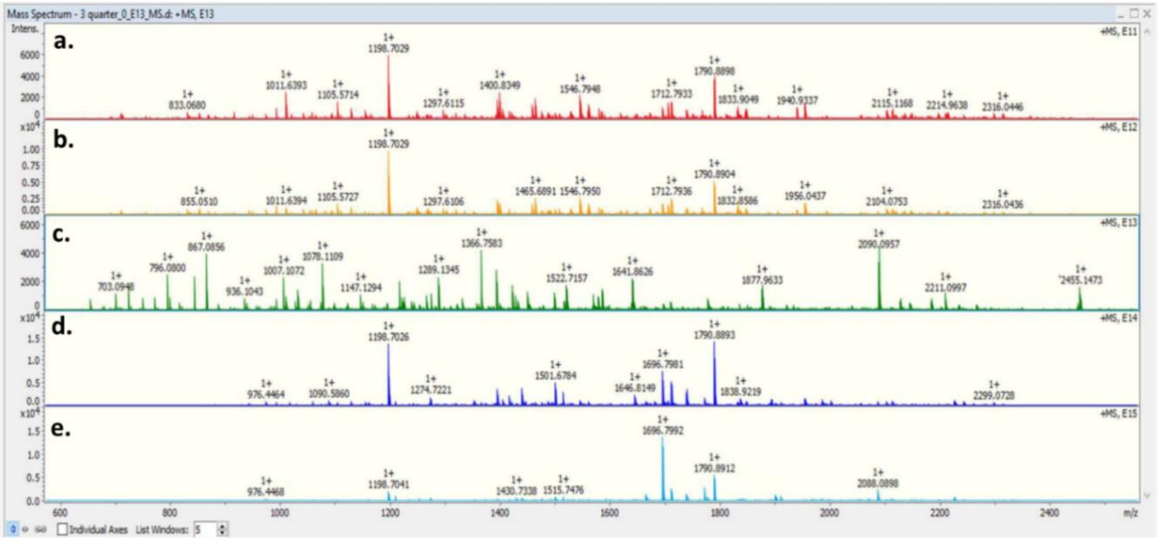

Figure S5. On-tissue trypsin digestion of FFPE heart tissue from **A.** Congo red positive (CR+) and **B.** Congo red negative (CR-) biopsy specimen; **C.** in-solution trypsin digestion of a transthyretin protein standard; **D.** Congo red positive (CR+) and **E.** Congo red negative (CR-) autopsy specimen.

Figure S6

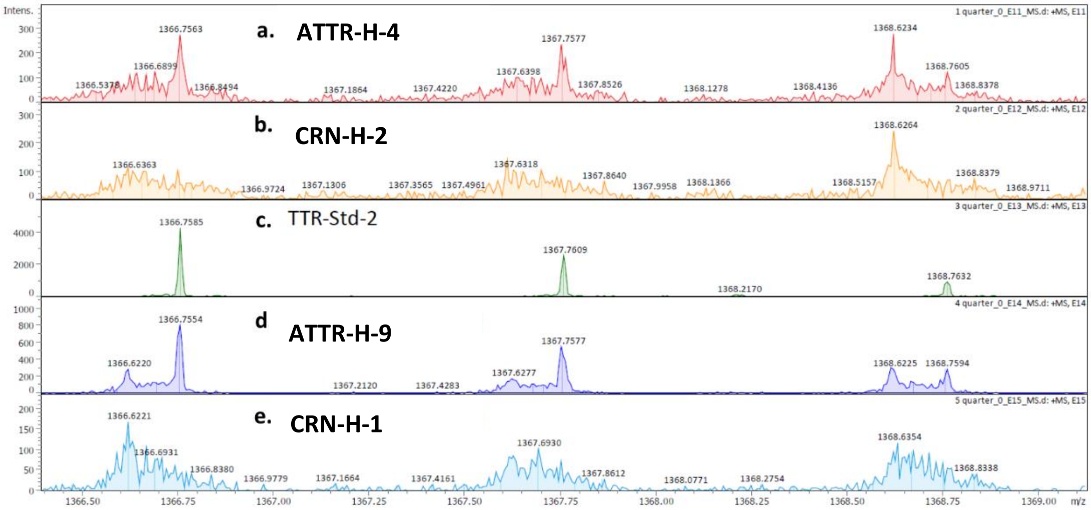

Figure S6. Peptide GSPAINVAVHVFR (m/z 1366.7) as potential biomarker for transthyretin observed only in **A.** Congo red positive heart biopsy (ATTR-H-4), **C.** transthyretin protein standard (TTR-Std-2) and **D.** Congo red positive heart autopsy (ATTR-H-9) but not in the **B.** Congo red negative heart biopsy (CRN-H-2) and **E.** Congo red negative heart autopsy specimen (CRN-H-1).

Figure S7

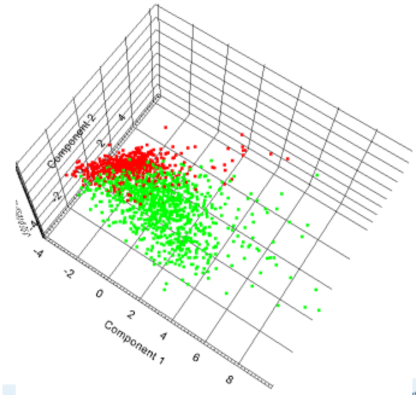

Fig. S7. Principal component analysis of Congo red negative biopsy specimen (CRN-H-2) versus Congo red positive specimen (ATTR-H-4).
