# Supplementary material for: Effect of dynamic exclusion and the use of FAIMS, DIA and MALDI-mass spectrometry imaging with ion mobility on amyloid protein identification
Source: Clin Proteomics. 2024 Jul 3;21:47. doi: 10.1186/s12014-024-09500-w (PMC11223398; doi:10.1186/s12014-024-09500-w)
Supplement: Supplementary file 2 — Supplementary Material 2. Table S1—Amyloidosis sub-types from tissue specimens from different organs analyzed by combined laser capture microdissection and liquid chromatography tandem mass spectrometry (LC-MS/MS). Table S5—Collision cross section (CCS) values from isobaric peptides observed from CR (+) and CR (−) tissues analyzed and resolved by MALDI-ultrahigh resolution and TIMS. Table S6—List of amyloidosis samples and the different platforms used for data acquisition. Table S7—Mass spectrometer parameters and settings for the Velos, Lumos and Exploris. Table S8—Cost comparison of amyloidosis sub-typing. Table S9—Cost analysis amyloidosis subtyping by MALDI-MSI. [file 12014_2024_9500_MOESM2_ESM.docx]

**Supplementary Tables**

**S1, S5, S6, S7, S8 and S9**

**Table S1.** Amyloidosis sub-types from tissue specimens from different organs analyzed

by combined laser capture microdissection and liquid chromatography tandem mass

spectrometry (LC-MS/MS)

**Table S5.** Collision cross section (CCS) values from isobaric peptides observed from CR (+) and CR (-) tissues analyzed and resolved by MALDI-ultrahigh resolution and TIMS.


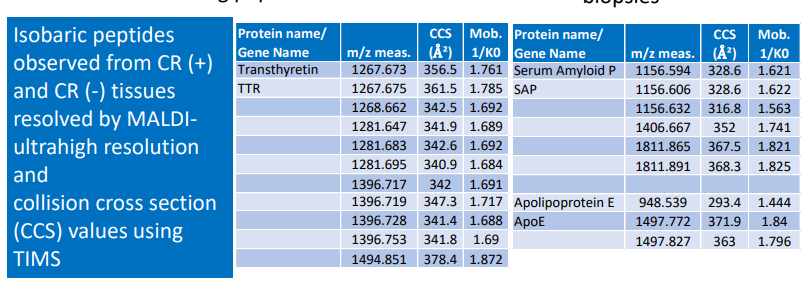


**Table S6.** List of amyloidosis samples and the different platforms used for data acquisition

**Table S7.** Mass spectrometer parameters and settings for the LTQ Orbitrap, Velos, Lumos and Exploris 480

**-----------------------------------------------------------------------------------------------------------------------------------------**

**LTQ VELOS**

MS Run Time (min): 60.00

Scan Event Details:

1: FTMS + p norm o(300.0-1600.0)

CV = 0.0V

2: ITMS + c norm Dep MS/MS Most intense ion from (1)

Activation Type: CID

Min. Signal Required: 20000.0

Isolation Width: 1.50

Normalized Coll. Energy: 35.0

Default Charge State: 2

Activation Q: 0.250

Activation Time: 30.000

CV = 0.0V

3: FTMS + p norm Dep MS/MS Most intense ion from (1)

Activation Type: CID

Min. Signal Required: 20000.0

Isolation Width: 1.50

Normalized Coll. Energy: 35.0

Default Charge State: 2

Activation Q: 0.250

Activation Time: 30.000

CV = 0.0V

Scan Event 2 and 3 repeated for top 3 peaks.

Lock Masses:

Pos List Name: N/A

Source: API Source

Mass List: (none)

Neg List Name: N/A

Source: API Source

Mass List: (none)

Data Dependent Settings:

Use separate polarity settings disabled

Parent Mass List: (none)

Reject Mass List: (none)

Neutral Loss Mass List: (none)

Product Mass List: (none)

Neutral loss in top: 3

Product in top: 3

Most intense if no parent masses found not enabled

Add/subtract mass not enabled

FT master scan preview mode enabled

Charge state screening enabled

Charge state dependent ETD time not enabled

Monoisotopic precursor selection not enabled

Non-peptide monoisotopic recognition not enabled

Charge state rejection enabled

Unassigned charge states : rejected

Charge state 1 : rejected

Charge state 2 : not rejected

Charge state 3 : not rejected

Charge states 4+ : not rejected

Chromatography mode is disabled

Global Data Dependent Settings:

Predict ion injection time enabled

Use global parent and reject mass lists not enabled

Exclude parent mass from data dependent selection not enabled

Exclusion mass width by mass

Exclusion mass width low: 1.50000

Exclusion mass width high: 1.50000

Parent mass width by mass

Parent mass width low: 0.50000

Parent mass width high: 0.50000

Reject mass width relative to mass

Reject mass width relative to low (ppm): 10.000

Reject mass width relative to high (ppm): 10.000

Zoom/UltraZoom scan mass width by mass

Zoom/UltraZoom scan mass width low: 5.00

Zoom/UltraZoom scan mass width high: 5.00

FT SIM scan mass width low: 5.00

FT SIM scan mass width high: 5.00

Neutral Loss candidates processed by decreasing intensity

Neutral Loss mass width by mass

Neutral Loss mass width low: 0.50000

Neutral Loss mass width high: 0.50000

Product candidates processed by decreasing intensity

Product mass width by mass

Product mass width low: 0.50000

Product mass width high: 0.50000

MS mass range: 0.00-1000000.00

MSn mass range by mass

MSn mass range: 0.00-1000000.00

Use m/z values as masses not enabled

Analog UV data dep. not enabled

Dynamic exclusion enabled

Repeat Count: 4

Repeat Duration: 10.00

Exclusion List Size: 500

Exclusion Duration: 20.00

Exclusion mass width by mass

Exclusion mass width low: 1.50000

Exclusion mass width high: 1.50000

Expiration: disabled

Isotopic data dependence not enabled

Custom Data Dependent Settings:

Not enabled

**Lumos**

DDA OTIT

MS Global Settings

Use Static Source Gasses

Use Ion Source Settings from Tune = Not Checked

Method Duration (min)= 73

Ion Source Type = NSI

Spray Voltage = Static

Spray Voltage: Positive Ion (V) = 2600

Spray Voltage: Negative Ion (V) = 2100

Gas Mode = Static

Ion Transfer Tube Temp (°C) = 275

FAIMS Mode = Not Installed

Application Mode = Peptide

Pressure Mode = Standard

Expected Peak Width (s) = 30

Default Charge State = 2

Experiment #1 [MS]

Start time (min): 2

End time (min): 73

Cycle Time (sec): 2

Master Scan

MS OT

Detector type Orbitrap

Resolution = 120,000

Mass range = Normal

Quadrupole Isolation = True

Scan Range (m/z) = 300-1200

RF Lens (%) = 60

AGC Target: Standard

Maximum Injection Time Mode: Custom

Maximum Injection Time (ms): 200

Microscans:1

Data Type: Centroid

Polarity: Positive

Source Fragmentation: Disabled

Scan Description:

Filters:

MIPS

Monoisotopic Peak Determination Peptide

Charge State

Include charge state(s): 2-5

Include undetermined charge state: False

Intensity

Filter Type = Intensity Threshold

Intensity Threshold: 1.0e4

Dynamic Exclusion

Exclude after n times = 1

Exclusion duration(s) 30

Mass Tolerance ppm

Low 10

High 10

Exclude Isotopes True

Perform dependent scan on single charge state per precursor only: False

Precursor Fit:

Data Dependent Mode: Cycle Time

Time between master scan(sec) = 2

Scan Event type 1:

Scane

ddMS^2^ IT HCD

Isolation Mode: Quadrupole

Isolation Window (m/z) : 1.6

Isolation Offset: Off

Activation type: HCD

Collision Energy Mode: Fixed

Collision Energy Type: Normalized

HCD Collision Energy (%): 35

Detector type: Ion trap

Ion trap Scan Rate: Rapid

Mass Range: Normal

ScanRange Mode: Auto

AGC Target: Standard

Maximum Injection Time Mode: Dynamic

Microscans: 1

Data Type: Centroid

Scan Description:

Start time (min) 0

End time (min) 2

Experiment #2 (MS)

Start time (min) 0

End time (min) 2

Master Scan

MS OT

Detector Type: Orbitrap

Orbitrap Resolution: 120000

Mass Range: Normal

Use Quadrupole Isolation: True

Scan Range (m/z) 300-1200

RF Lens (5) 30

AGC Target Custom

Normalized AGC Target (%) 50

Maximum Injection Rime (ms) 50

Microscan 1

Data Type Profile

Polarity Negtaive

Source Fragmentation Disabled

**Exploris 480**

**DDA No FAIMS with no Dynamic Exclusion**

Method Summary

Method Settings

Application Mode: Peptide

Method Duration (min): 73

Global Parameters

Ion Source

Ion Source Type: NSI

Spray Voltage: Static

Positive Ion (V): 2700

Negative Ion (V): 2000

Ion Transfer Tube Temp (°C): 275

Use Ion Source Settings from Tune: False

FAIMS Mode: Not Installed

MS Global Settings

Infusion Mode: Liquid Chromatography

Expected LC Peak Width (s): 30

Advanced Peak Determination: True

Default Charge State: 2

Enable Xcalibur AcquireX Ab method modifications: False

Internal Mass Calibration: Off

Experiment #1 [MS]

Start Time (min): 0

End Time (min): 2

Master Scan:

Full Scan

Orbitrap Resolution: 120000

Scan Range (m/z): 250-1200

RF Lens (%): 50

AGC Target: Standard

Maximum Injection Time Mode: Auto

Microscans: 1

Data Type: Centroid

Polarity: Negative

Source Fragmentation: Disabled

Scan Description:

Experiment #2 [FAIMS-NOT-INSTALLED]

Start Time (min): 2

End Time (min): 73

Master Scan:

Full Scan

Orbitrap Resolution: 120000

Scan Range (m/z): 300-1200

RF Lens (%): 50

AGC Target: Custom

Normalized AGC Target (%): 100

Maximum Injection Time Mode: Auto

Microscans: 1

Data Type: Centroid

Polarity: Positive

Source Fragmentation: Disabled

Scan Description: -50

Filters:

MIPS

Monoisotopic peak determination: Peptide

Relax restrictions when too few precursors are found: True

Intensity

Filter Type: Intensity Threshold

Intensity Threshold: 1.0e4

Charge State

Include charge state(s): 2-6

Include undetermined charge states: False

Precursor Fit

Fit Threshold (%): 70

Fit Window (m/z): 1.6

Data Dependent

Data Dependent Mode: Cycle Time

Time between Master Scans (sec): 1

Scan Event Type 1:

Scan:

ddMS²

Multiplex Ions: False

Isolation Window (m/z): 1.6

Isolation Offset: Off

Collision Energy Type: Normalized

HCD Collision Energies (%): 30

Orbitrap Resolution: 7500

TurboTMT: Off

Scan Range Mode: Auto

AGC Target: Standard

Maximum Injection Time Mode: Auto

Microscans: 1

Data Type: Centroid

**DDA No FAIMS with Dynamic Exclusion**

Method Summary

Method Settings

Application Mode: Peptide

Method Duration (min): 73

Global Parameters

Ion Source

Ion Source Type: NSI

Spray Voltage: Static

Positive Ion (V): 2700

Negative Ion (V): 2000

Ion Transfer Tube Temp (°C): 275

Use Ion Source Settings from Tune: False

FAIMS Mode: Not Installed

MS Global Settings

Infusion Mode: Liquid Chromatography

Expected LC Peak Width (s): 30

Advanced Peak Determination: True

Default Charge State: 2

Enable Xcalibur AcquireX Ab method modifications: False

Internal Mass Calibration: Off

Experiment #1 [MS]

Start Time (min): 0

End Time (min): 2

Master Scan:

Full Scan

Orbitrap Resolution: 120000

Scan Range (m/z): 250-1200

RF Lens (%): 50

AGC Target: Standard

Maximum Injection Time Mode: Auto

Microscans: 1

Data Type: Centroid

Polarity: Negative

Source Fragmentation: Disabled

Scan Description:

Experiment #2 [FAIMS-NOT-INSTALLED]

Start Time (min): 2

End Time (min): 73

Master Scan:

Full Scan

Orbitrap Resolution: 120000

Scan Range (m/z): 300-1200

RF Lens (%): 50

AGC Target: Custom

Normalized AGC Target (%): 100

Maximum Injection Time Mode: Auto

Microscans: 1

Data Type: Centroid

Polarity: Positive

Source Fragmentation: Disabled

Scan Description: -50

Filters:

MIPS

Monoisotopic peak determination: Peptide

Relax restrictions when too few precursors are found: True

Intensity

Filter Type: Intensity Threshold

Intensity Threshold: 1.0e4

Charge State

Include charge state(s): 2-6

Include undetermined charge states: False

Precursor Fit

Fit Threshold (%): 70

Fit Window (m/z): 1.6

Dynamic Exclusion

Dynamic Exclusion Mode: Auto

Data Dependent

Data Dependent Mode: Cycle Time

Time between Master Scans (sec): 1

Scan Event Type 1:

Scan:

ddMS²

Multiplex Ions: False

Isolation Window (m/z): 1.6

Isolation Offset: Off

Collision Energy Type: Normalized

HCD Collision Energies (%): 30

Orbitrap Resolution: 7500

TurboTMT: Off

Scan Range Mode: Auto

AGC Target: Standard

Maximum Injection Time Mode: Auto

Microscans: 1

Data Type: Centroid

**DDA FAIMS with no Dynamic Exclusion**

Orbitrap Exploris 480 Method Summary

Global Settings

Use Static Source Gasses

Use Ion Source Settings from Tune = Not Checked

Method Duration (min)= 73

Ion Source Type = NSI

Spray Voltage = Static

Spray Voltage: Positive Ion (V) = 2700

Spray Voltage: Negative Ion (V) = 2000

Gas Mode = Static

Infusion Mode (LC)= False

Ion Transfer Tube Temp (°C) = 275

APPI Lamp = Not in use

Total Carrier Gas Flow = 4.6

FAIMS Mode = User Defined

FAIMS Inner Electrode Temp = 100

FAIMS Outer Electrode Temp = 100

Application Mode = Peptide

Pressure Mode = Standard

Expected Peak Width (s) = 30

Default Charge State = 2

Advanced Peak Determination = True

Xcalibur AcquireX enabled for method modifications = False

Experiment 1

Experiment Name = MS

Start Time (min) = 0

End Time (min) = 2

Cycle Time (sec) = 3

Scan MasterScan

Orbitrap Resolution = 120000

Scan Range (m/z) = 250-1200

Microscans = 1

AGC Target = Standard

RF Lens(%) = 50

Maximum Injection Time Mode = Auto

FAIMS Voltages On = True

DataType = Centroid

FAIMS CV = 0

Polarity = Negative

Source Fragmentation = False

Scan Description =

Experiment 2

Experiment Name = MS-50

Start Time (min) = 2

End Time (min) = 73

Cycle Time (sec) = 1

Scan MasterScan

Orbitrap Resolution = 120000

Scan Range (m/z) = 300-1200

Normalised AGC Target (%)= 100

Microscans = 1

Maximum Injection Time Mode = Auto

AGC Target = Custom

RF Lens(%) = 50

DataType = Centroid

FAIMS Voltages On = True

FAIMS CV = -50

Polarity = Positive

Source Fragmentation = False

Scan Description = -50

Filter MIPS

Filter Type = MIPS

Filter Label =

MIPS Mode = Peptide

Relax Restrictions when too few Precursors are Found = True

Filter IntensityThreshold

Filter Type = IntensityThreshold

Filter Label =

Intensity Filter Type = IntensityThreshold

Minimum Intensity = 10000

Filter ChargeState

Filter Type = ChargeState

Filter Label =

Include charge state(s) = 2-6

Include undetermined charge states = False

Filter Purity

Filter Type = Purity

Filter Label =

Purity Threshold (%) = 70

Purity Window = 1.6

Data Dependent Properties

Data Dependent Mode= Cycle Time

Scan Event 1

Scan ddMSnScan

Isolation Window (m/z) = 1.6

Isolation Window = Custom

Multiplex Ions Enabled = False

Isolation Offset = Off

Reported Mass = Offset Mass

Maximum number of multiplexed ions = 0

Collision Energy Type = Normalized

HCD Collision Energies (%) = 30

Orbitrap Resolution = 7500

Microscans = 1

Maximum Injection Time Mode = Auto

AGC Target = Standard

DataType = Centroid

Source Fragmentation = False

Scan Description =

Experiment 3

Experiment Name = MS-70

Start Time (min) = 2

End Time (min) = 73

Cycle Time (sec) = 1

Scan MasterScan

Orbitrap Resolution = 120000

Scan Range (m/z) = 300-1200

Normalised AGC Target (%)= 100

Microscans = 1

Maximum Injection Time Mode = Auto

AGC Target = Custom

RF Lens(%) = 50

DataType = Centroid

FAIMS Voltages On = True

FAIMS CV = -70

Polarity = Positive

Source Fragmentation = False

Scan Description = -70

Filter MIPS

Filter Type = MIPS

Filter Label =

MIPS Mode = Peptide

Relax Restrictions when too few Precursors are Found = True

Filter IntensityThreshold

Filter Type = IntensityThreshold

Filter Label =

Intensity Filter Type = IntensityThreshold

Minimum Intensity = 10000

Filter ChargeState

Filter Type = ChargeState

Filter Label =

Include charge state(s) = 2-6

Include undetermined charge states = False

Filter Purity

Filter Type = Purity

Filter Label =

Purity Threshold (%) = 70

Purity Window = 1.6

Data Dependent Properties

Data Dependent Mode= Cycle Time

Scan Event 1

Scan ddMSnScan

Isolation Offset = Off

Multiplex Ions Enabled = False

Isolation Window (m/z) = 1.6

Isolation Window = Custom

Maximum number of multiplexed ions = 0

Reported Mass = Offset Mass

Collision Energy Type = Normalized

HCD Collision Energies (%) = 30

Orbitrap Resolution = 7500

Microscans = 1

Maximum Injection Time Mode = Auto

AGC Target = Standard

DataType = Centroid

Source Fragmentation = False

Scan Description = -70

**DDA FAIMS with Dynamic exclusion**

Exploris 480

Orbitrap Exploris 480 Method Summary

Global Settings

Use Static Source Gasses

Use Ion Source Settings from Tune = Not Checked

Method Duration (min)= 73

Ion Source Type = NSI

Spray Voltage = Static

Spray Voltage: Positive Ion (V) = 2700

Spray Voltage: Negative Ion (V) = 2000

Gas Mode = Static

Infusion Mode (LC)= False

Ion Transfer Tube Temp (°C) = 275

APPI Lamp = Not in use

Total Carrier Gas Flow = 4.6

FAIMS Mode = User Defined

FAIMS Inner Electrode Temp = 100

FAIMS Outer Electrode Temp = 100

Application Mode = Peptide

Pressure Mode = Standard

Expected Peak Width (s) = 30

Default Charge State = 2

Advanced Peak Determination = True

Xcalibur AcquireX enabled for method modifications = False

Experiment 1

Experiment Name = MS

Start Time (min) = 0

End Time (min) = 2

Cycle Time (sec) = 3

Scan MasterScan

Orbitrap Resolution = 120000

Scan Range (m/z) = 250-1200

Microscans = 1

AGC Target = Standard

RF Lens(%) = 50

Maximum Injection Time Mode = Auto

FAIMS Voltages On = True

DataType = Centroid

FAIMS CV = 0

Polarity = Negative

Source Fragmentation = False

Scan Description =

Experiment 2

Experiment Name = MS-50

Start Time (min) = 2

End Time (min) = 73

Cycle Time (sec) = 1

Scan MasterScan

Orbitrap Resolution = 120000

Scan Range (m/z) = 300-1200

Normalised AGC Target (%)= 100

Microscans = 1

Maximum Injection Time Mode = Auto

AGC Target = Custom

RF Lens(%) = 50

DataType = Centroid

FAIMS Voltages On = True

FAIMS CV = -50

Polarity = Positive

Source Fragmentation = False

Scan Description = -50

Filter MIPS

Filter Type = MIPS

Filter Label =

MIPS Mode = Peptide

Relax Restrictions when too few Precursors are Found = True

Filter IntensityThreshold

Filter Type = IntensityThreshold

Filter Label =

Intensity Filter Type = IntensityThreshold

Minimum Intensity = 10000

Filter ChargeState

Filter Type = ChargeState

Filter Label =

Include charge state(s) = 2-6

Include undetermined charge states = False

Filter Purity

Filter Type = Purity

Filter Label =

Purity Threshold (%) = 70

Purity Window = 1.6

Dynamic Exclusion

Share dynamic exclusion list with other selected dynamic exclusion filters: False

Dynamic Exclusion Mode: Auto

Data Dependent

Data Dependent Mode: Cycle Time

Time between Master Scans (sec): 1

Scan Event 1

Scan ddMSnScan

Isolation Window (m/z) = 1.6

Isolation Window = Custom

Multiplex Ions Enabled = False

Isolation Offset = Off

Reported Mass = Offset Mass

Maximum number of multiplexed ions = 0

Collision Energy Type = Normalized

HCD Collision Energies (%) = 30

Orbitrap Resolution = 7500

Microscans = 1

Maximum Injection Time Mode = Auto

AGC Target = Standard

DataType = Centroid

Source Fragmentation = False

Scan Description =

Experiment 3

Experiment Name = MS-70

Start Time (min) = 2

End Time (min) = 73

Cycle Time (sec) = 1

Scan MasterScan

Orbitrap Resolution = 120000

Scan Range (m/z) = 300-1200

Normalised AGC Target (%)= 100

Microscans = 1

Maximum Injection Time Mode = Auto

AGC Target = Custom

RF Lens(%) = 50

DataType = Centroid

FAIMS Voltages On = True

FAIMS CV = -70

Polarity = Positive

Source Fragmentation = False

Scan Description = -70

Filter MIPS

Filter Type = MIPS

Filter Label =

MIPS Mode = Peptide

Relax Restrictions when too few Precursors are Found = True

Filter IntensityThreshold

Filter Type = IntensityThreshold

Filter Label =

Intensity Filter Type = IntensityThreshold

Minimum Intensity = 10000

Filter ChargeState

Filter Type = ChargeState

Filter Label =

Include charge state(s) = 2-6

Include undetermined charge states = False

Filter Purity

Filter Type = Purity

Filter Label =

Purity Threshold (%) = 70

Purity Window = 1.6

Dynamic Exclusion

Share dynamic exclusion list with other selected dynamic exclusion filters: False Dynamic Exclusion Mode: Auto

Data Dependent Data Dependent Mode: Cycle Time

Time between Master Scans (sec): 1

Scan Event 1

Scan ddMSnScan

Isolation Offset = Off

Multiplex Ions Enabled = False

Isolation Window (m/z) = 1.6

Isolation Window = Custom

Maximum number of multiplexed ions = 0

Reported Mass = Offset Mass

Collision Energy Type = Normalized

HCD Collision Energies (%) = 30

Orbitrap Resolution = 7500

Microscans = 1

Maximum Injection Time Mode = Auto

AGC Target = Standard

DataType = Centroid

Source Fragmentation = False

Scan Description = -70

**DIA no FAIMS**

Orbitrap Exploris 480 Method Summary

Global Settings

Use Static Source Gasses

Use Ion Source Settings from Tune = Not Checked

Method Duration (min)= 28

Ion Source Type = NSI

Spray Voltage = Static

Spray Voltage: Positive Ion (V) = 2600

Spray Voltage: Negative Ion (V) = 2100

Gas Mode = Static

Infusion Mode (LC)= False

Ion Transfer Tube Temp (°C) = 275

APPI Lamp = Not in use

FAIMS Mode = Not Installed

Application Mode = Peptide

Pressure Mode = Standard

Expected Peak Width (s) = 30

Default Charge State = 2

Advanced Peak Determination = True

Xcalibur AcquireX enabled for method modifications = False

Experiment 1

Experiment Name = MS

Start Time (min) = 2

End Time (min) = 28

Cycle Time (sec) = 3

Scan MasterScan

Orbitrap Resolution = 120000

Scan Range (m/z) = 350-1100

Time (ms) = 45

Normalised AGC Target (%)= 300

Microscans = 1

AGC Target = Custom

RF Lens(%) = 60

Maximum Injection Time Mode = Custom

DataType = Centroid

Polarity = Positive

Source Fragmentation = False

Scan Description =

Experiment 2

Experiment Name = MS

Start Time (min) = 0

End Time (min) = 2

Cycle Time (sec) = 3

Scan MasterScan

Orbitrap Resolution = 120000

Scan Range (m/z) = 250-1200

Time (ms) = 100

Normalised AGC Target (%)= 10

Microscans = 1

Maximum Injection Time Mode = Custom

AGC Target = Custom

RF Lens(%) = 30

DataType = Profile

Polarity = Negative

Source Fragmentation = False

Scan Description =

Experiment 3

Experiment Name = DIA - 50 SCAN EVENTS and FAIMS NOT INSTALLED

Start Time (min) = 2

End Time (min) = 28

Cycle Time (sec) = 3

Scan DIAScan

Isolation Window (m/z) = 15

Isolation Window = Custom

Window Overlap (m/z) = 1

Multiplex Ions Enabled = False

Window Placement Optimization = On

Number Of Scan Events = 50

HCD Collision Energies (%) = 33

Collision Energy Type = Normalized

Orbitrap Resolution = 7500

Precursor MassRange (m/z) = 350-1100

Time (ms) = 22

Normalised AGC Target (%)= 1000

Microscans = 1

Maximum Injection Time Mode = Custom

AGC Target = Custom

Scan Range Mode = Auto

DataType = Centroid

Polarity = Positive

Source Fragmentation = False

Time Mode = Start/End Time

Scan Description =

>>>>>>>>>>>>> Mass List Table <<<<<<<<<<<<<<

m/z|Calculated m/z Window|

357.5|349.40870775-365.41598375|

372.5|364.415529-380.422805|

387.5|379.42235025-395.42962625|

402.5|394.4291715-410.4364475|

417.5|409.43599275-425.44326875|

432.5|424.442814-440.45009|

447.5|439.44963525-455.45691125|

462.5|454.4564565-470.4637325|

477.5|469.46327775-485.47055375|

492.5|484.470099-500.477375|

507.5|499.47692025-515.48419625|

522.5|514.4837415-530.4910175|

537.5|529.49056275-545.49783875|

552.5|544.497384-560.50466|

567.5|559.50420525-575.51148125|

582.5|574.5110265-590.5183025|

597.5|589.51784775-605.52512375|

612.5|604.524669-620.531945|

627.5|619.53149025-635.53876625|

642.5|634.5383115-650.5455875|

657.5|649.54513275-665.55240875|

672.5|664.551954-680.55923|

687.5|679.55877525-695.56605125|

702.5|694.5655965-710.5728725|

717.5|709.57241775-725.57969375|

732.5|724.579239-740.586515|

747.5|739.58606025-755.59333625|

762.5|754.5928815-770.6001575|

777.5|769.59970275-785.60697875|

792.5|784.606524-800.6138|

807.5|799.61334525-815.62062125|

822.5|814.6201665-830.6274425|

837.5|829.62698775-845.63426375|

852.5|844.633809-860.641085|

867.5|859.64063025-875.64790625|

882.5|874.6474515-890.6547275|

897.5|889.65427275-905.66154875|

912.5|904.661094-920.66837|

927.5|919.66791525-935.67519125|

942.5|934.6747365-950.6820125|

957.5|949.68155775-965.68883375|

972.5|964.688379-980.695655|

987.5|979.69520025-995.70247625|

1002.5|994.7020215-1010.7092975|

1017.5|1009.70884275-1025.71611875|

1032.5|1024.715664-1040.72294|

1047.5|1039.72248525-1055.72976125|

1062.5|1054.7293065-1070.7365825|

1077.5|1069.73612775-1085.74340375|

1092.5|1084.742949-1100.750225|

>>>>>>>>>>>>> End Mass List Table <<<<<<<<<<<<<<

**Table S8.** Cost analysis amyloidosis subtyping by LMD/LC-MS/MS

**Table S9.** Cost analysis amyloidosis subtyping by MALDI-MSI
